# A Proprioception-Centered Hypothesis Linking Sensorimotor Function, Respiration, and Sleep Across the Day–Night Cycle in Developmental Dyslexia

**DOI:** 10.3390/brainsci16101106

**Published:** 2026-10-14

**Authors:** Patrick Quercia, Steeve Morin

**Affiliations:** 1 CAPS UMR 1093, INSERM, Université Bourgogne Europe, F-21000 Dijon, France; 2 Cabinet de Podolologie, F-64120 Amendeuix-Oneix, France; morin.steeve@neuf.fr

**Keywords:** developmental dyslexia, proprioception, sensorimotor coherence, embodied cognition, motor control, sleep-disordered breathing, sleep–wake cycle, neuroplasticity, multisensory integration, child sleep

## Abstract

**Highlights:**

**What are the main findings?**

- A proprioception-centered sensorimotor coherence framework is proposed to relate motor regulation, spatial localization, multisensory integration, respiration, and sleep across the day–night cycle in developmental dyslexia.
- A clinical examination based on functional responses to sensory modulation is used to characterize sensorimotor organization and to identify convergent regulatory effects across functional domains.

**What are the implications of the main findings?**

- The framework provides a complementary embodied perspective linking daytime sensorimotor organization with nighttime physiological conditions relevant to recovery, plasticity, and learning.
- The convergence of clinical and experimental observations provides a rationale for further testing the potential role of proprioception-centered sensorimotor coherence in developmental dyslexia.

**Abstract:**

**Objectives:** Developmental dyslexia is a heterogeneous neurodevelopmental condition in which motor, perceptual, attentional, respiratory, and sleep-related differences have been reported alongside reading difficulties. This Perspective proposes a proprioception-centered sensorimotor coherence framework to consider potential interactions among these domains across the day–night cycle. **Methods:** This hypothesis-generating narrative Perspective traces the progressive development of the proposed framework from clinical observations to experimental investigations and subsequent refinement of a clinical examination based on functional responses to sensory modulation. No systematic review or new cohort analysis was undertaken. The examination explores three principal domains—tonic-motor regulation, spatial localization, and multisensory integration—together with respiratory and sleep-related factors. Sensorimotor modulation includes ocular, oral, plantar, respiratory, manual, and ergonomic approaches selected according to their observed functional effects across domains. A clinical case illustrates the application of this approach. **Results:** Experimental studies conducted over nearly two decades in our INSERM laboratory objectively investigated postural regulation, proprioceptive acuity, spatial localization, multisensory processing, and action representation in developmental dyslexia. More recent exploratory clinical studies extended the investigation to respiration and sleep. Taken together, these observations suggest that proprioception-centered sensorimotor coherence may represent one functional level through which motor, perceptual, respiratory, attentional, and learning-related processes interact across the day–night cycle as learning develops and continues. **Conclusions:** The proposed framework is a working hypothesis rather than an established causal model or a validated treatment for developmental dyslexia. It provides a complementary embodied perspective and identifies clinically observable and experimentally testable relationships that warrant further investigation, including through controlled studies examining the contributions of individual sensory and functional components.

## 1. Introduction: From Postural Control to Developmental Dyslexia

Developmental dyslexia is traditionally defined as a specific disorder of reading acquisition occurring despite normal intelligence, adequate schooling, and the absence of identified sensory or neurological deficits [1]. For several decades, phonological models have dominated explanations of dyslexia, emphasizing impairments in speech-sound processing and difficulties in establishing stable associations between phonemes and graphemes.

These models have substantially improved our understanding of the mechanisms involved in reading acquisition. However, the marked clinical heterogeneity observed among children with dyslexia suggests that multiple mechanisms may contribute to the expression of the disorder. Numerous studies have reported motor, postural, attentional, and perceptual differences in some children with dyslexia that are not fully accounted for by strictly linguistic models [2,3,4].

Among these associated manifestations, disturbances of postural control occupy a particular place. Beginning in the 1970s, several authors reported abnormalities in balance regulation and body orientation in some children with dyslexia. These observations led Frank and Levinson to propose a vestibulo-cerebellar contribution to dyslexia, while Nicolson and colleagues later developed the cerebellar deficit hypothesis, suggesting impairments in the automatization of both motor and cognitive processes [5,6].

During the same period, the Portuguese physician Henrique Martins da Cunha proposed an original clinical approach based on systematic observation [7]. He described a coherent constellation of postural, motor, perceptual, and cognitive signs observed in some children with learning difficulties, which he grouped under the term Postural Deficiency Syndrome. His hypothesis was that disturbances of postural regulation could have consequences extending beyond motor function and might influence cognitive performance.

Although this approach remained largely marginal within cognitive neuroscience for many years, recent advances in the understanding of postural control mechanisms have renewed interest in these observations [8]. Postural regulation is now recognized as far more than a simple balance-maintenance function. Unlike many specialized physiological systems, postural control does not depend on a single sensory modality. Instead, it requires the continuous integration of visual, vestibular, somatosensory, trigeminal, and respiratory information, together with the mechanical constraints imposed by the environment [9,10,11].

From this perspective, posture can be viewed as the visible expression of an ongoing process that continuously reconciles multiple streams of sensory information. When these signals converge, the system can generate stable and energy-efficient motor organization. When they become inconsistent or insufficiently integrated, adaptive strategies may emerge to preserve balance and spatial orientation.

This property makes postural control particularly informative about multisensory integration. Beyond its motor function, it provides a window into the interactions between perception, action, and cognition. Postural organization may therefore provide information not only about how an individual maintains balance, but also about how the nervous system coordinates, prioritizes, and integrates the diverse sensory information continuously reaching it. This view is consistent with the theoretical framework of embodied cognition, according to which cognitive functions emerge from ongoing interactions between the brain, the body, and the environment [12]. In a related perspective, Berthoz proposed that perception and action rely on the continuous construction of internal reference frames integrating sensory and motor information [13].

Within this conceptual framework, alterations in sensorimotor organization may affect the stability of body-centered spatial reference frames and may thereby be associated with changes in posture, spatial localization, attention, respiration, sleep, or learning. Importantly, the present article does not assume that such relationships constitute an established causal pathway. Rather, it examines whether convergent clinical and experimental observations can support a testable hypothesis concerning the interaction of these functions.

Within this framework, reading acquisition depends not only on linguistic processes but also on the efficiency of sensorimotor mechanisms supporting visuospatial attention, spatial representation, and multisensory integration [14,15]. Over the past two decades, a series of experimental studies have explored relationships between proprioception, postural control, spatial localization, and multisensory integration in children with developmental dyslexia [16,17,18,19,20,21,22,23]. These studies have reported sensorimotor differences associated with alterations in postural regulation, perceptual stability, and multisensory processing. More recently, investigations into respiratory function and sleep have raised the possibility that some of these phenomena may be embedded within a broader dynamic involving the entire sleep–wake cycle [24].

The purpose of the present article is not to provide a systematic or comprehensive review of these experimental findings, which have recently been summarized elsewhere [25]. Instead, this Perspective describes how initial clinical observations, subsequent experimental investigations, and accumulated clinical experience progressively generated questions concerning proprioception, sensorimotor organization, respiration, and sleep in children with developmental dyslexia.

In the present article, sensorimotor coherence is used as a functional, hypothesis-generating framework rather than as a validated psychometric construct or a directly measurable clinical variable. It refers to the functional consistency observed between sensory information, motor responses, and the body-centered spatial reference frames involved in perception and action. The concept is not intended to represent a single latent trait, composite score, or diagnostic threshold.

The three principal functional domains considered in this framework are tonic-motor regulation, spatial localization, and multisensory integration. These domains are treated as complementary areas of clinical and experimental investigation rather than as independent diagnostic tests or quantitative indicators of a single underlying construct. Their relationships provide a basis for examining how different sensory conditions may influence sensorimotor organization in an individual child.

The clinical approach described in this Perspective therefore seeks not to identify a single sign or to establish a diagnosis from one test, but to examine how sensorimotor responses change when defined sensory conditions are modified. The resulting observations are interpreted in the context of the child’s overall clinical presentation and are intended to generate hypotheses concerning the sensory factors that may contribute to the observed organization.

The article subsequently presents the principles of this clinical examination, the experimental findings that contributed to the development of the hypothesis, the sensorimotor modulation strategies derived from these observations, and their possible relationship with respiratory function and the sleep–wake cycle. To illustrate how this clinical reasoning is applied in practice, a single clinical case is presented as a pedagogical example, allowing the reader to follow the sequence of observations and the iterative process of assessment that underlies the approach. This case is not intended to be representative of children with developmental dyslexia, nor does it constitute evidence of diagnostic validity, therapeutic efficacy, or causal mechanisms. Throughout the article, established findings, experimental observations, clinical observations, and physiological hypotheses are distinguished explicitly.

## 2. Posture as an Expression of Sensorimotor Organization

As discussed above, postural control relies on the continuous integration of information originating from multiple sensory systems. From this perspective, posture can be regarded as an observable expression of the way in which these sensory signals are integrated within the sensorimotor system [9,10].

An important question nevertheless remains: do all sensory inputs contribute equally to this organization?

Clinical experience, observations associated with Postural Deficiency Syndrome, and experimental investigations conducted over the last several decades suggest that some sensory modalities may occupy a more strategic position than others.

Among the sensory systems involved in postural regulation, proprioception appears to play a particularly important role [8,26]. Whereas certain afferent systems primarily provide information about the external environment or about body orientation in space, proprioception continuously informs the central nervous system about the state of the body itself, including segmental position, muscle tension, and ongoing movement [11,27,28].

This function gives proprioception an important role in establishing a body-centered reference frame within which other sensory signals can be interpreted and integrated. Such an internal reference may contribute to the consistency between perception and action.

This potential role had already been anticipated in the original descriptions of Postural Deficiency Syndrome. Martins da Cunha associated the syndrome with disturbances of both visual and proprioceptive functions and interpreted the observed motor, perceptual, and cognitive manifestations as different expressions of a common sensorimotor regulatory dysfunction [7].

For many years, however, this hypothesis remained essentially clinical. Available methods allowed investigators to observe the postural and perceptual consequences of a possible proprioceptive dysfunction, but did not allow proprioceptive performance to be assessed directly.

This situation changed with the development of experimental paradigms capable of evaluating proprioceptive acuity using tasks that do not directly require reading. Using passive low-velocity limb movement detection tasks, studies conducted in our laboratory demonstrated that children with developmental dyslexia exhibited, on average, lower proprioceptive acuity than typically developing readers [29]. More importantly, proprioceptive performance was significantly correlated with both reading skills and phonological abilities.

These findings do not establish a simple causal relationship between proprioception and dyslexia. They nevertheless indicate that differences in proprioceptive performance can be objectively observed using tasks that do not directly require reading, although they do not establish whether such differences are primary, secondary, or related to other developmental characteristics associated with dyslexia. They also provide a basis for the hypothesis that the postural, motor, perceptual, and multisensory differences reported in some children with dyslexia may be related to alterations in sensorimotor regulation.

Within this framework, posture should not be viewed merely as a secondary consequence of cognitive difficulties. Rather, it may provide a clinically accessible window onto the organization of the sensorimotor system and its adaptation to sensory conditions. Its study can provide information about motor, perceptual, attentional, and adaptive processes that may interact during learning.

This integrative perspective forms the basis of the clinical approach presented in the present article. Rather than attempting to correct isolated postural, visual, or respiratory abnormalities, the clinician seeks to identify sensory and sensorimotor conditions that modify the individual’s functional organization and to use the observed responses to generate hypotheses about potentially relevant adaptive processes.

The experimental studies conducted over the past two decades were not designed to establish a new theory of developmental dyslexia. Their primary contribution has been to show that several longstanding clinical observations can be objectively measured and experimentally investigated. Consequently, the clinical question is not simply whether sensorimotor differences may exist in some children with dyslexia, but how such observations can be identified, interpreted, and investigated further within an individualized clinical assessment.

## 3. From Clinical Observation to Experimental Investigation: Progressive Development of a Proprioception-Centered Sensorimotor Coherence Framework

### 3.1. From Clinical Observations to Experimental Questions

The clinical observations briefly introduced above progressively led to a series of questions extending beyond routine clinical practice. Did the postural characteristics observed in some children with developmental dyslexia reflect specific features of sensorimotor organization? Could some of the perceptual phenomena reported by patients be objectively demonstrated under experimental conditions? More generally, could relationships exist between postural control, spatial perception, multisensory processing, and learning?

The studies presented below emerged from these successive questions. They were not initially designed to test a predefined theory, but rather to determine whether recurring clinical observations could be objectively measured and experimentally investigated.

One of the distinctive features of the original description of Postural Deficiency Syndrome was the proposed involvement of both visual and proprioceptive information processing [7]. This clinical hypothesis raised a fundamental question: could the contribution of proprioception be demonstrated experimentally?

This question was particularly relevant because proprioception contributes to the construction of body-centered and spatial reference frames. The experimental work described below therefore progressively examined several functions in which proprioceptive information could potentially contribute spatial localization, multisensory integration, and proprioceptive acuity.

### 3.2. Experimental Investigation of Motor Control, Spatial Localization, Multisensory Processing, and Proprioceptive Acuity

The first experimental studies focused on postural and motor control, extending the initial clinical observations into objectively measurable parameters. In children with developmental dyslexia, clinical observations had consistently identified abnormalities of postural organization and signs interpreted within the framework of proprioceptive dysfunction [16,17]. Subsequent force-platform studies provided an objective assessment of these observations and demonstrated differences in postural control between children with developmental dyslexia and typically developing readers, particularly under conditions requiring greater postural stability [18].

Further studies examined whether postural control was influenced by cognitive demand and proprioceptive integration. When attention was engaged by a concurrent cognitive task, children with developmental dyslexia showed increased postural activity, indicating that cognitive demands could interfere with the regulation of posture [19]. This effect was reduced in children who had undergone proprioceptive intervention, suggesting that some aspects of sensorimotor organization remained modifiable. Subsequent experiments using proprioceptive perturbations further demonstrated altered integration of proprioceptive signals during postural control in children with developmental dyslexia. These responses were also subject to improvement following proprioceptive intervention [22]. Taken together, these studies suggested that the postural differences observed clinically were not simply static motor abnormalities, but could involve altered integration of proprioceptive information under changing sensory and cognitive conditions.

The investigation was then extended to spatial localization. The conceptual advances introduced by Alain Berthoz and the pioneering experimental work of Jean-Pierre Roll contributed to a broader understanding of spatial perception and action. Berthoz emphasized the active construction of internal representations linking the body, sensory information, and future action [13]. Within this perspective, perception is not simply a passive registration of sensory inputs but involves the continuous construction and updating of spatial reference frames.

These theoretical developments converged with the experimental studies of Roll, who demonstrated that proprioceptive signals arising from muscles, joints, and extraocular muscles contribute to the construction of spatial reference frames used to localize objects and guide action [30,31,32,33]. Spatial localization therefore depends not only on visual information but also on the integration of proprioceptive information concerning body position and movement.

**Figure 1:**
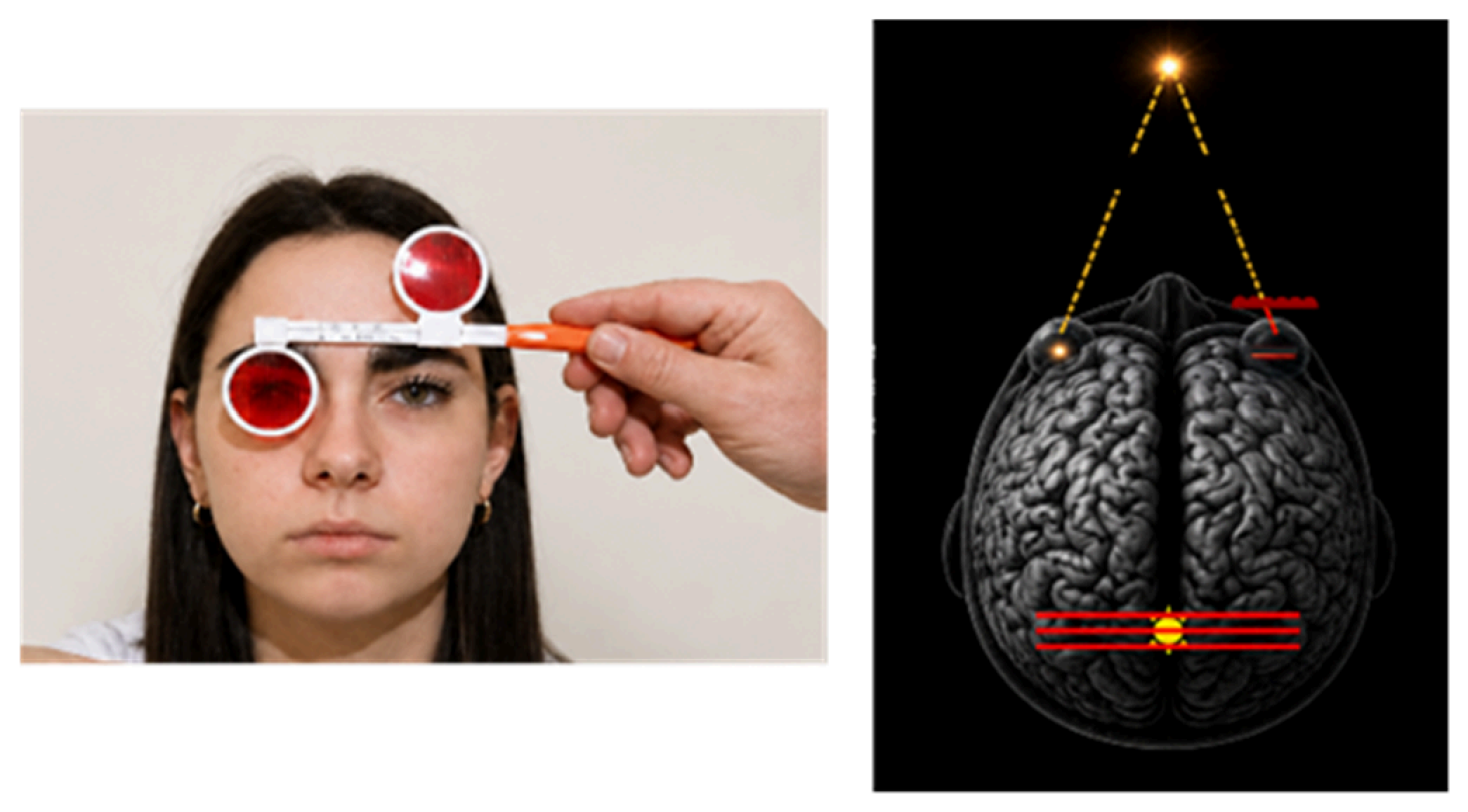
A calibrated point light source (0.7 mm diameter, 100 lumens, positioned 3 m from the child) is viewed through a Maddox rod consisting of vertically aligned high-power cylindrical prisms. The Maddox rod converts the point source into a horizontal red line for one eye, whereas the fellow eye continues to perceive the point light. The relative position of the red line with respect to the light source is used to assess binocular visual alignment and spatial localization.

This perspective provided a rationale for investigating spatial localization in children with developmental dyslexia. Matheron had previously used the Maddox test in patients with Postural Deficiency Syndrome to investigate relationships between visual instability and chronic pain (Figure 1) [34]. Building upon this work, we developed adaptations of the procedure for children with developmental dyslexia [35] (Supplementary Materials: Video S1: Perceptual Maddox Test).

The Vertical Maddox Test was used to explore binocular spatial localization and its modulation by different sensory inputs. The principle of the test and its application in the present clinical examination are illustrated in the accompanying video. The physiological rationale and experimental basis of this paradigm have been described in detail in a previous study investigating the role of the Maddox test in the assessment of postural control [36].

The Maddox paradigm was of particular interest because it introduces a controlled sensory conflict between different sources of information contributing to spatial localization. Our studies investigated the stability of perceived visual alignment under conditions designed to increase the relative contribution of ocular proprioceptive information. The observations obtained with this paradigm suggested differences in visual spatial localization in some children with developmental dyslexia under these experimental conditions [35].

These findings provided a basis for further investigating whether similar phenomena could be observed when other sensory modalities were involved. Experimental paradigms subsequently revealed transient disturbances of visual perception in children with developmental dyslexia during conditions designed to increase the influence of ocular proprioceptive information [37]. These phenomena consisted of transient partial disappearances of specific regions of the visual field during auditory stimulation. We termed these experimentally induced perceptual phenomena “pseudoscotomas,” by analogy with visual field defects observed in ophthalmological and neurological disorders. Unlike true scotomas, however, these phenomena were transient, reversible, and dependent on the experimental conditions.

Similar effects could also be induced through proprioceptive manipulation by vibration of postural or non-postural muscles. This observation suggested that the phenomenon might not be limited to auditory–visual interactions and raised the possibility that proprioceptive perturbation could influence multisensory perceptual organization more broadly [37].

The physiological mechanisms underlying these observations remain unresolved. The findings nevertheless provided an experimental basis for examining relationships among proprioceptive information, motor organization, spatial perception, and multisensory processing.

A further question was whether proprioceptive performance itself could be assessed directly. Experimental paradigms based on the detection of extremely slow passive limb movements made it possible to evaluate proprioceptive acuity using tasks that did not directly require reading [29]. Using these methods, our group observed lower proprioceptive acuity, on average, in children with developmental dyslexia compared with typically developing readers. Proprioceptive performance was also significantly correlated with reading ability and certain phonological skills.

These findings do not establish that proprioceptive dysfunction causes dyslexia. They indicate, however, that differences in proprioceptive performance can be objectively observed using tasks that do not directly require reading, although they do not establish whether such differences are primary, secondary, or related to other developmental characteristics associated with dyslexia [29].

The experimental investigation was subsequently extended from motor control and proprioceptive processing to the internal representation of action. In adolescents with developmental dyslexia, studies of actual and mentally simulated movements identified differences in the temporal organization of action representation, and the quality of motor imagery was related to reading and phonological performance [24]. These findings extended the sensorimotor perspective beyond postural regulation and proprioceptive acuity toward the representation of action itself.

Taken together, these experimental observations provided convergent information across several functional domains: motor control, proprioceptive integration and acuity, spatial localization, multisensory processing, and action representation. They did not establish a common causal mechanism, nor did they demonstrate that proprioceptive dysfunction is the primary cause of developmental dyslexia. They nevertheless provided an experimental basis for reconsidering the relationships among these domains and for examining whether they could be understood within a broader framework of sensorimotor organization.

### 3.3. From Convergent Observations to a Proprioception-Centered Sensorimotor Coherence Framework

The convergence of these clinical and experimental observations progressively led us to reconsider how the different findings might be related. Posture, spatial localization, multisensory processing, and proprioceptive acuity could no longer be considered entirely in isolation. Their potential interactions suggested the usefulness of a broader functional framework centered on proprioception.

It was in this context that the concept of proprioception-centered sensorimotor coherence emerged. This concept does not refer to a validated psychometric construct, a directly measurable clinical variable, or a diagnostic entity. Rather, it provides a functional, hypothesis-generating framework for describing the coherence that may emerge from the continuous interaction between proprioceptive information and other sensory and motor processes.

Within this framework, proprioception is not considered to provide a complete explanation for developmental dyslexia, nor to replace cognitive, linguistic, phonological, or attentional models. Rather, it is considered one component of a broader integrative framework in which sensorimotor organization may interact with these domains during development.

This framework also provides a possible interpretation of sensorimotor, postural, perceptual, respiratory, and sleep-related manifestations that are often considered comorbid features of developmental dyslexia. Rather than assuming that these manifestations are necessarily independent comorbidities, the model explores whether some of them may represent interconnected expressions of an altered sensorimotor organization developing over time.

Within this framework, three functional domains are particularly relevant to the clinical exploration of sensorimotor coherence: motor control, spatial localization, and multisensory integration. Together, they form the three vertices of the dysproprioceptive sensorimotor coherence triangle. The triangle is conceptual rather than a measurement model: it provides a framework for organizing clinical observations and generating experimentally testable hypotheses concerning the interactions among these domains.

The framework would be weakened if these domains failed to show reproducible relationships, if the observed relationships could not be distinguished from those expected from measurement variability or relevant confounding factors, or if they failed to provide explanatory information beyond established cognitive, linguistic, attentional, or motor characteristics. These questions cannot be resolved within the present Perspective and will require prospective experimental studies using appropriate quantitative and psychometric methods.

### 3.4. Extending the Clinical–Experimental Framework to Respiration and Sleep

The proprioception-centered sensorimotor coherence framework subsequently led us to consider whether sensorimotor organization might also interact with respiratory function and the sleep–wake cycle.

During the clinical follow-up of children presenting with proprioceptive disorders, fatigue was one of the most frequently reported symptoms. For many years, this fatigue was primarily interpreted in relation to increased muscle tone and the compensatory effort required to maintain postural stability. However, systematic interviews with parents of children with developmental dyslexia progressively drew attention to another recurring pattern: restless sleep, frequent awakenings, non-restorative sleep, and persistent morning fatigue. These observations were consistent with previous reports suggesting a possible relationship between sleep disturbances and learning processes [38,39,40,41].

At the same time, clinical examination repeatedly revealed paradoxical respiratory patterns and altered thoraco-abdominal coordination, suggesting abnormal diaphragmatic involvement. These observations prompted us to investigate whether respiratory function might be related to the sensorimotor organization observed during wakefulness.

This question was informed by the work of Gandevia, who emphasized the contribution of the diaphragm to postural control [42]. Beyond its ventilatory function, the diaphragm contributes to trunk stabilization and anticipatory postural adjustments. We therefore considered the possibility that altered sensorimotor organization might modify the balance between its respiratory and postural roles.

A recurring clinical observation was an apparently heel-weighted stance, despite considerable variability in the visible postural configurations of the children. Rather than interpreting this finding solely as a biomechanical abnormality, we considered whether it might reflect an alteration in the body-centered spatial reference frame. In this interpretation, the perceived vertical could be slightly shifted backward, leading to compensatory strategies to maintain postural stability. The resulting postural organization could involve greater recruitment of the diaphragm for postural stabilization and, potentially, a modification of its respiratory function.

A further clinical observation contributed to the development of this hypothesis. Subtle signs of sensorimotor dysfunction were also observed in some siblings who did not present significant reading difficulties. This discrepancy prompted us to look for factors that might influence the clinical expression of sensorimotor alterations. Sleep assessment revealed no major sleep disturbance in these children, whereas a relatively high proportion of their parents were receiving continuous positive airway pressure (CPAP) at a comparatively young age. Although these observations were not designed to establish epidemiological differences, they raised the possibility that respiratory and sleep-related factors might modulate the expression of sensorimotor alterations and contributed to our subsequent interest in sleep.

These observations subsequently motivated the development of protocols specifically dedicated to sleep assessment in children with developmental dyslexia [24]. In an exploratory series, overnight recordings showed respiratory abnormalities compatible with increased upper-airway resistance, including elevated respiratory effort-related arousals and frequent inspiratory flow limitation. These findings were compatible with the sleep-related breathing disorder described by Guilleminault and colleagues as upper airway resistance syndrome (UARS) [43]. However, the observations did not establish that UARS is characteristic or specific of developmental dyslexia.

The sleep findings therefore provided a further clinical and experimental question rather than a confirmation of the proposed framework. Could subtle respiratory disturbances during sleep represent one of the factors through which daytime sensorimotor organization interacts with nocturnal recovery and, ultimately, with learning processes?

The extension of the investigation from posture, spatial localization, multisensory integration, and proprioceptive acuity to respiration and sleep therefore represents a further development of the clinical hypothesis rather than its confirmation. The proposed relationships between daytime sensorimotor organization, respiratory regulation, sleep, and learning remain to be independently tested.

## 4. Clinical Examination of Sensorimotor Coherence

The clinical approach presented in this article is based on a simple premise: sensorimotor coherence cannot be observed directly. It can only be inferred from the functional responses observed across several domains and from the way these responses change when sensory conditions are modified.

The observations and experimental findings described in the previous sections progressively led to this approach. Studies of postural and motor control, spatial localization, multisensory integration, proprioceptive acuity, respiration, and sleep suggested that no single sensory or motor function was sufficient to account for the range of phenomena observed in some children with developmental dyslexia. The clinically relevant question therefore shifted from identifying an isolated abnormality to examining how different functional domains respond to controlled changes in sensory conditions.

The clinical examination derived from this framework is not intended as a psychometric test battery or as a set of independent diagnostic tests. It consists of a structured sequence of clinical observations in which each functional domain is explored in turn. Within each domain, the clinician characterizes the observed response and examines whether specific sensory conditions regulate, perturb, or leave the function unchanged.

For example, during the Perceptual Maddox Test, the clinician evaluates variations in perceived line position under different sensory conditions. The same principle is applied to the other functional domains, using the procedures appropriate to each one. The purpose is not simply to determine whether a function is abnormal, but to characterize its individual response pattern to sensory modulation.

The responses obtained within each domain are then compared across domains. Particular attention is paid to whether the same sensory condition produces concordant or divergent effects. For example, a sensory stimulation that modifies spatial localization may become clinically informative if the same stimulation also improves motor control or reduces the occurrence of a perceptual phenomenon such as a pseudoscotoma.

Within this framework, sensorimotor coherence refers to the consistency of functional responses across domains, rather than to the mere existence of statistical or conceptual relationships between them. Concordant responses may strengthen an individualized working hypothesis, whereas divergent responses may indicate that an observed effect is more specific to a particular functional domain.

This approach also provides a basis for evaluating potential therapeutic adjustments. A sensory configuration is considered more coherent when it produces favorable and concordant effects across several functional domains, rather than improving one domain while leaving another unchanged or producing a perturbing response elsewhere. The same functional examination can subsequently be repeated during follow-up to determine whether the observed changes are maintained, reproduced, or modified.

**Figure 2:**
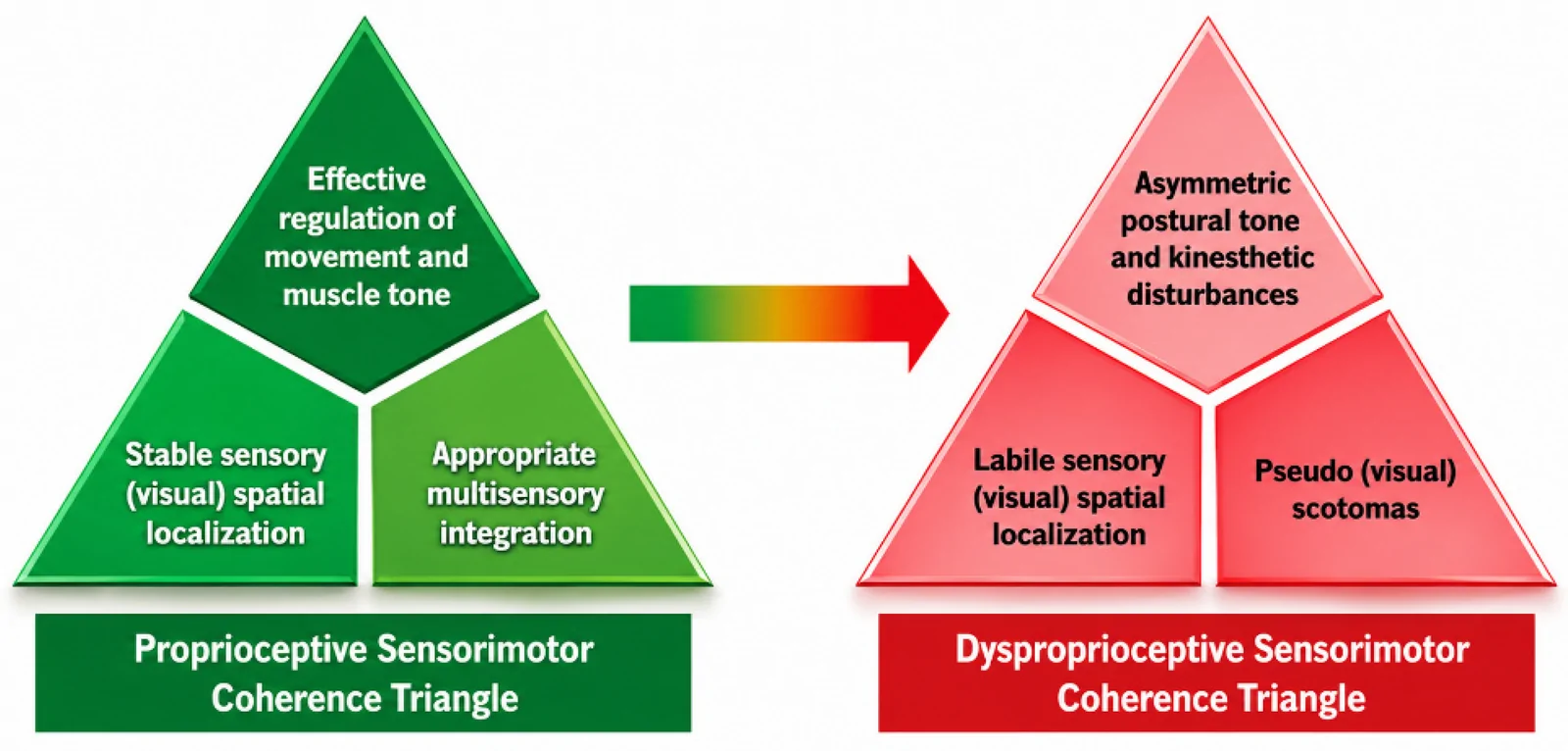
Conceptual representation of the dysproprioceptive sensorimotor coherence triangle as a clinical pathway. The three vertices correspond to tonic-motor regulation, spatial localization, and multisensory integration, which are explored sequentially and then compared across sensory conditions. The triangle is intended primarily to organize clinical assessment and guide individualized sensory modulation, rather than to serve as a diagnostic or quantitative model.

The three principal functional domains explored in this clinical approach can be represented schematically as the dysproprioceptive sensorimotor coherence triangle (Figure 2). Its three vertices correspond to motor control, spatial localization, and multisensory integration. The triangle is not intended to represent a validated measurement model or to imply that these three domains constitute a single latent construct. Rather, it provides a conceptual and organizational framework for structuring the clinical examination and comparing the responses obtained across domains.

The triangle therefore serves primarily as a clinical pathway: first to characterize each functional domain, then to compare the effects of the same sensory conditions across domains, and finally to evaluate whether a proposed sensory adjustment produces a coherent response throughout the system. It is consequently a conceptual framework rather than a diagnostic instrument or a validated clinical score. Its reproducibility, the relationships between its different domains, and its clinical utility remain to be established through prospective studies using appropriate quantitative and psychometric methods.

### 4.1. Structured Clinical History

The assessment systematically begins with a structured clinical history based on a questionnaire developed through a multicentre study involving ten independent centres and ten clinicians from different regions of France (Table 1) [44].

Its development and statistical analysis were conducted with methodological support from CENBIOTECH, a specialised biostatistical research organisation in Dijon, France. The questionnaire was subsequently evaluated in 243 children, including 109 children with developmental dyslexia and 134 typically developing readers [44].

The questionnaire was designed as a structured clinical inquiry rather than as a stand-alone diagnostic instrument. Its purpose is to systematically explore potentially relevant manifestations before the clinical examination and to help identify the functional domains requiring more detailed investigation.

It explores four domains:

- manifestations related to tonic-motor regulation;
- difficulties involving spatial localization;
- perceptual and multisensory manifestations;
- signs suggestive of sleep-related disturbances.

The sleep-related questions incorporate some items derived from or informed by the Sleep Disturbance Scale for Children (SDSC), a validated questionnaire developed by Bruni et al. [45]. The questionnaire as a whole is a distinct clinical instrument and should not be considered a reproduction of the SDSC.

Questions are distributed between the child and the parents. Parents report observable nighttime manifestations, while the child is questioned about subjective daytime experiences. For sleep-related items, the questionnaire is administered by the clinician after parents have observed their child during several hours of sleep.

Each item is scored on a five-point frequency scale: 1 = Never, 2 = Occasionally (1 or 2 times a month), 3 = Sometimes (1 or 2 times a week), 4 = Often (3 to 5 times a week), and 5 = Every day. Higher scores therefore indicate a higher reported frequency of the corresponding manifestation. The total score is obtained by summing the scores of the 34 items.

The questionnaire underwent substantial statistical characterization during its development, including item reduction, assessment of internal consistency, principal component analysis, and receiver operating characteristic (ROC) analysis. The overall standardized Cronbach’s alpha was 0.88, and the ROC analysis yielded an area under the curve of 0.891 [44]. Exploratory score thresholds were also examined in the original study population. Scores ≥ 80 were associated with a markedly different distribution between children with developmental dyslexia and typically developing readers. These thresholds were derived from the original study population and were not established as diagnostic cut-offs.

In the present clinical approach, the questionnaire score is therefore not used to establish the diagnosis or severity of dysproprioception. Its primary function is to organize the clinical history, identify clusters of potentially relevant manifestations, and guide the subsequent clinical examination. The clinician does not base the clinical interpretation on the questionnaire score alone, but uses its findings to determine which functional domains and sensory conditions require further investigation.

The questionnaire may also provide a structured reference for longitudinal follow-up. Repeating the same questions may help identify changes in reported manifestations over time, although such changes remain clinical observations and should not be considered a validated quantitative measure of treatment efficacy. The questionnaire should therefore be regarded as a structured and statistically characterized clinical inquiry that guides, but does not replace, the clinical examination. Its external validity, diagnostic specificity, and test–retest reliability remain to be established in independently recruited populations.

The questionnaire was administered directly by the clinician to the parents and to the child, and the responses were recorded during the clinical interview. The content and structure of the instrument used in our clinical studies have been preserved in Table 1; only minor linguistic corrections were made to the English wording without changing the intended meaning of the items.

### 4.2. Postural Observation: A First Reading of Sensorimotor Organization

Postural observation generally constitutes the first step of the clinical examination because it provides an immediate view of the way the child organizes body orientation and support in the upright position.

The purpose of this observation is not to identify a characteristic postural phenotype of developmental dyslexia. Clinical experience shows considerable variability in the visible organization of posture from one child to another. The clinician therefore does not seek to match the child to a predefined postural archetype, but rather to identify recurrent features that may provide an initial indication of the underlying sensorimotor organization.

Two observations have been particularly consistent in our clinical experience. The first is the presence of a functional scoliotic attitude, usually visible in the frontal plane and variable in its expression. The second is a predominant or maximal heel loading, observed during standing assessment and particularly apparent on plantar-pressure examination (see Section 4.7).

These two observations are considered together rather than as isolated abnormalities. A functional scoliotic attitude may reflect an asymmetry of postural tone, whereas predominant heel loading raises a different question: whether the child’s internal reference for body orientation and support may be altered. Within the framework proposed here, excessive heel loading is therefore considered a possible clinical expression of an anteroposterior postural imbalance, potentially related to altered sensorimotor organization, rather than simply as a mechanical characteristic of the foot.

This interpretation remains a hypothesis rather than a direct demonstration of altered proprioceptive perception. Heel loading is not specific to proprioceptive dysfunction and may be influenced by biomechanical, vestibular, visual, or other postural factors. Its clinical interest therefore lies less in its isolated presence than in its relationship with the responses obtained during the subsequent functional examination.

Other postural features may also be observed, including asymmetry of the shoulder or pelvic girdles, head rotation or lateral inclination, sagittal imbalance, anterior projection of the head, or modifications of respiratory abdominal mobility. None of these findings is considered diagnostic, and none is required to define the clinical profile. They contribute to the overall observation and may provide additional clues concerning the strategies adopted by the child to organize postural control.

The diversity of postural organizations observed in children with developmental dyslexia is therefore important in itself. The common feature is not a particular posture, but the possibility that different postural configurations may represent different adaptive solutions to an altered sensorimotor reference. This distinction is central to the clinical approach described in this Perspective.

Postural observation consequently provides an initial orientation rather than a diagnostic conclusion. It allows the clinician to formulate preliminary questions that are subsequently tested dynamically: is the observed asymmetry stable or modifiable? Is the predominant heel loading altered when sensory conditions change? Do these changes correspond to modifications in other functional domains? The answers to these questions are sought through the subsequent examination of tonic-motor regulation, spatial localization, and multisensory integration.

### 4.3. Dynamic Motor Tests

Static postural observation provides an initial view of the child’s sensorimotor organization, but it does not by itself distinguish between an underlying tonic asymmetry and the adaptive strategy that has developed around it. The examination therefore continues with a series of dynamic clinical manoeuvres designed to modify, temporarily and in a controlled manner, selected sensory conditions and to observe the resulting changes in motor organization.

These manoeuvres have a dual purpose. They first characterize the observed motor organization by determining whether a response is normal or abnormal and whether it is stable or modifiable under defined sensory or postural conditions. When an abnormal response is identified, different sensory modulations, used individually or in combination, can then be tested to determine whether they regulate, leave unchanged, or further perturb the response. This provides clinical information about the functional organization of the response and preliminary guidance for the subsequent sensorimotor modulation strategy.

The dynamic examination includes several complementary procedures. Among them, cephalic rotations and extensions are used to explore the response of tonic organization to changes in head position, while the Podal Convergence Manoeuvres provide a more specific exploration of changes in the resistance of the hip external rotators under defined sensory conditions. The responses obtained during these procedures can subsequently be compared with findings from spatial localization and multisensory integration testing, allowing the clinician to determine whether similar sensory conditions produce concordant effects across functional domains.

These procedures remain clinical observations rather than validated psychometric or biomechanical measurements, and their reproducibility and objective correlates require further investigation. Their clinical role, however, extends beyond the identification of an abnormal motor response: by revealing whether and under which sensory conditions that response can be modified, they contribute to the formulation of an individualized sensorimotor hypothesis and to the selection of subsequent therapeutic interventions.

#### 4.3.1. Head Rotations and Extensions

The cephalic region occupies a central position in postural regulation. It brings together visual, vestibular, oculomotor, trigeminal, and cervical information that contributes to the spatial reference frames used to orient the body in space [9].

The range of cephalic rotation and extension provides a simple clinical means of assessing the symmetry of the tonic-motor organization. Under normal conditions, these movements are expected to be approximately symmetrical.

When a tonic asymmetry is present, without presupposing its origin, the range of rotation or extension may be shorter on the ipsilateral or contralateral side (see Section 4.7). The direction of this asymmetry is clinically relevant because it provides an initial indication of the side on which a slightly stronger ocular sensory modulation may subsequently need to be explored.

The cephalic examination therefore provides an early indication of the ocular sensory modulations that may need to be explored during the subsequent clinical examination.

#### 4.3.2. Podal Convergence Maneuvers (MCPs)

The Podal Convergence Maneuvers (MCPs, from the French Manœuvres de Convergence Podale) are clinical dynamic maneuvers used to explore changes in tonic-motor organization under defined sensory or physiological conditions. Their primary observable outcome is a change or absence of change in the resistance perceived during passive movement of the hip external rotators.

The term “hypertonia” used in the description of the MCPs therefore refers to an increase in perceived resistance during the maneuver and does not imply a quantitatively established increase in muscle stiffness, muscle activity, or resting muscle tone. The examiner is principally looking for an on–off response: whether the resistance perceived at baseline changes when a defined condition is introduced.

The MCPs are not intended to reproduce the child’s habitual posture or to provide an isolated measurement of muscle tone. Rather, they temporarily modify selected sensory, postural, or respiratory conditions and allow the examiner to determine whether the tonic response is physiologically appropriate. Three variants are used in clinical practice, each exploring a different set of conditions.

➢ ***MCP 2D: Exploration of cephalic sensory inputs***

MCP 2D successively modifies several sources of information originating from the cephalic region, including oculomotor, oral, cervical, and temporomandibular inputs (see Section 4.7).

The maneuver begins with a defined baseline condition, with the gaze in primary position and the hands placed on the umbilicus. The examiner assesses the resistance of the hip external rotators and then repeats the assessment after each specified sensory modification. The clinical question is not the absolute level of resistance, but whether the resistance changes when the sensory condition is modified. A response may therefore be classified clinically as regulatory, neutral, or perturbing according to whether the perceived resistance decreases, remains essentially unchanged, or increases relative to baseline.

Because the outcome is based on the examiner’s perception of resistance, MCP 2D should not be considered a quantitative measure of muscle tone. Its clinical value lies in the reproducibility and consistency of the observed response across the predefined sequence and in its comparison with responses obtained in other functional domains.

➢ ***MCP 3D: Exploration of Proprioceptive Directional Coding***

MCP 3D explores the response of tonic organization to changes in upper-limb position in three-dimensional space (see Section 4.7). Because proprioceptive information contributes to the directional coding of limb position, a symmetrical and opposite mobilization of the upper limbs would not be expected, under physiological conditions, to produce an asymmetrical tonic response of the hip external rotators.

The upper limbs are therefore placed successively in standardized positions, thereby modifying the proprioceptive configuration of the body. Particular attention is paid to the presence or absence of a change in perceived resistance and to the reproducibility of the response, rather than to small quantitative differences in resistance.

These observations are interpreted within the clinical framework in which the MCPs are routinely used. The physiological mechanisms underlying the observed responses, however, remain to be objectively investigated.

➢ ***MCP Rescue: Exploration of Respiration–Swallowing–Posture Interactions***

The MCP Rescue explores the interaction between respiratory and swallowing conditions and tonic-motor organization (see Section 4.7).

The maneuver is performed with the eyes closed and after a brief period of relaxation intended to minimize voluntary compensatory responses. The examiner first establishes the baseline response of the hip external rotators and then observes whether this response changes under defined respiratory and swallowing conditions.

These conditions may include thoracic breathing, abdominal breathing, transient respiratory restriction, and swallowing following each of the respiratory conditions. Each respiratory condition is tested in three successive trials. The clinical question is whether these conditions produce a reproducible change, or absence of change, in the perceived resistance of the hip external rotators.

The rationale for this examination is that breathing and swallowing are normally highly automated physiological functions, while their interaction with tonic-motor organization may provide information about the coordination of respiratory, oral, and postural functions. When a respiratory or swallowing condition produces a marked and reproducible change in the tonic response, this observation may indicate an interaction between these functional systems. The present clinical procedure does not, however, establish the physiological mechanism responsible for such a response.

When three slow abdominal breaths reduce a tonic asymmetry induced by another respiratory pattern or by swallowing, this response provides a clinical argument for exploring the restoration of effective abdominal breathing as part of the therapeutic strategy. Within the present framework, such a response may also support the hypothesis that improving diaphragmatic breathing during wakefulness could contribute to more appropriate respiratory organization during sleep, including rapid eye movement (REM) sleep.

The MCP Rescue is an awake clinical maneuver performed with the eyes closed. It is not intended to simulate REM sleep or to constitute a physiological model of sleep-disordered breathing. Its possible relationship with nocturnal respiratory or sleep-related phenomena remains to be investigated independently.

##### Clinical Interpretation of the MCPs

The MCPs are therefore used as clinical response tests rather than as validated measures of muscle tone. Their primary purpose is to determine whether the observed tonic response is physiologically appropriate under the conditions tested and, when it is not, to identify sensory conditions that may regulate or normalize the response. The interpretation is strengthened when a modulation identified during an MCP is concordant with findings obtained elsewhere in the examination. For example, a sensory condition that normalizes an abnormal tonic response may subsequently be examined for its effect on spatial localization or on the perceptual phenomena explored by the Perceptual Maddox Test.

Conversely, a response that is restricted to a single domain should be interpreted more cautiously. The purpose of the MCPs is therefore not to establish a diagnosis from a particular response, but to contribute to the iterative clinical process in which sensory conditions are tested successively and their effects compared across functional domains.

The reproducibility, inter-rater and intra-rater reliability, and objective physiological correlates of the MCPs have not yet been formally established. Future validation studies should determine whether the clinical responses obtained during these maneuvers correspond to measurable changes in muscle activity, force, movement, or other physiological variables, using methods such as dynamometry, electromyography, and motion analysis.

### 4.4. Spatial Localization: Exploring the Stability of Spatial Reference Frames

Following the assessment of tonic and motor regulation, the clinical examination proceeds to the second vertex of the proposed sensorimotor coherence framework: spatial localization.

Maintaining a stable representation of space requires the continuous integration of visual, vestibular, trigeminal, plantar, and proprioceptive information. Under ordinary conditions, these sources contribute to a sufficiently stable representation of object location and body orientation.

Experimental studies have suggested that some children with developmental dyslexia may exhibit increased instability of spatial localization under conditions that increase reliance on proprioceptive information [35]. The objective of the clinical examination is therefore not simply to evaluate ocular alignment, but to explore the stability of the spatial reference frame under defined sensory conditions.

For this purpose, we use a clinical adaptation of the Maddox test, referred to as the Perceptual Maddox Test [35]. The physiological principles underlying the procedure have been described previously (see Section 4.7 and Supplementary Materials: Video S1: Perceptual Maddox Test). Briefly, the test creates a condition in which conventional binocular retinal fusion is reduced while usual oculomotor compensatory mechanisms are constrained. Under these conditions, the perceived relationship between the luminous reference and the Maddox line may provide indirect information about the contribution of extraocular muscle proprioception to spatial localization.

The Perceptual Maddox Test is therefore used as a functional sensory challenge rather than as a conventional test of ocular alignment. The clinician observes how the perceived relationship between the line and the reference light changes when specific sensory conditions are successively modified.

The principal clinical information is not the absolute displacement observed under a single condition, but the stability of the perceived position across the different conditions. In some children, the perceived position of the line remains relatively stable; in others, it may vary substantially. We refer to this variability descriptively as spatial-localization lability.

This lability should not be interpreted as a specific diagnostic marker of developmental dyslexia or as evidence of a defined proprioceptive disorder. Rather, it constitutes a clinical observation whose significance is assessed in relation to the other findings obtained during the examination. The magnitude of the observed displacement is small and, in our reference study, remained consistently below 0.75 prism diopters. Displacements exceeding this threshold may instead reflect a conventional vertical phoria rather than the spatial-localization lability investigated by the Perceptual Maddox Test. Because the displacements are small, accurate assessment requires careful calibration of the fixation target so that the perceived line remains as thin as possible.

In our clinical procedure, the responses obtained under the different sensory conditions can be summarized by a Lability Index. This index is used primarily as a within-subject descriptive measure of response variability. Its clinical meaning should not be equated with a validated diagnostic threshold, as its normative values, diagnostic accuracy, sensitivity, specificity, and test–retest reliability have not yet been established independently.

The clinical interest of the Perceptual Maddox Test therefore lies in identifying how spatial localization responds to defined sensory conditions. A condition may reduce spatial lability, have little apparent effect, or increase variability. These response patterns are interpreted together with the findings obtained in the other functional domains and may contribute to the individualized clinical hypothesis developed during the examination.

### 4.5. Multisensory Integration: Exploring Perceptual Stability

After the assessment of tonic and motor regulation and spatial localization, the clinical examination proceeds to the third vertex of the dysproprioceptive sensorimotor coherence triangle: multisensory integration [37] (see Section 4.7).

The assessment is performed under the standardized conditions of the Perceptual Maddox Test. After the baseline response has been established, binaural auditory stimulation is introduced, and the child is asked to report any interruption or gap in the perceived red line. This provides a defined condition in which transient perceptual disturbances can be explored.

The examination then proceeds by modifying specific sensory inputs, including spinal proprioceptive, oral, and plantar stimulation. Under each condition, the clinician records whether a pseudoscotoma appears, disappears, increases, decreases, or remains unchanged. A stimulation may therefore have a perturbing, regulatory, or neutral effect on the perceptual response.

The term pseudoscotoma is used descriptively for this transient perceptual phenomenon and does not imply an organic visual-field defect. Its clinical interest lies primarily in the response to defined sensory manipulations and in the consistency of these responses across repeated conditions.

Experimental work has shown that extraocular muscle proprioception can influence visuospatial attention under specific sensory-conflict conditions [46]. These findings support the plausibility of interactions between proprioceptive and visual processes, while not establishing the specific mechanisms underlying the clinical phenomena observed here. The perceptual responses obtained during this assessment are subsequently compared with the findings from tonic-motor and spatial-localization testing. A sensory condition that produces concordant effects across domains may strengthen the individualized working hypothesis, whereas divergent effects suggest that the response may be more domain specific. These findings may then contribute to the selection of sensory modulations explored during the subsequent therapeutic assessment.

### 4.6. Sleep Assessment: Objective Evaluation of Nocturnal Respiratory Function

The clinical questionnaire provides an initial description of sleep-related symptoms and nocturnal behaviours. However, subjective observations alone cannot characterize the physiological nature or severity of sleep disturbances. When clinically indicated, objective assessment is therefore used to investigate nocturnal respiratory function and to determine whether further specialized evaluation is warranted.

Polysomnography remains the reference standard for the comprehensive assessment of sleep-disordered breathing. In our exploratory work, polysomnography was performed in 18 children with developmental dyslexia and without an attentional disorder. The recordings showed frequent respiratory effort-related arousals (RERAs), with a mean RERA index of 18.2 ± 7.9, together with a mean apnea–hypopnea index (AHI) of 3.6 ± 3.5 and frequent inspiratory flow limitation [24]. These observations were considered compatible with increased upper-airway resistance and contributed to the formulation of a specific clinical and physiological question concerning sleep-disordered breathing in this population. They do not, however, establish the prevalence or specificity of upper airway resistance syndrome (UARS) in developmental dyslexia.

Because access to polysomnography may be limited in routine clinical practice, we have also used JAWAC (JAW Activity) as a complementary ambulatory approach for the exploration and follow-up of nocturnal respiratory phenomena [47,48]. The technique records mandibular movements and micromovements during sleep using non-invasive sensors positioned on the forehead and in the submental region. Changes in mandibular activity provide indirect information related to respiratory effort and respiratory events and may identify patterns compatible with increased upper-airway resistance. The JAWAC signal itself, however, does not establish a diagnosis of UARS.

An important distinction must be made between parameters derived from JAWAC and conventional polysomnographic indices. The REI-JAWAC (Respiratory Effort Index issue from JAW activity) for example, is calculated from the mandibular signal alone and provides an indirect estimate of respiratory events per hour. It should not be equated with the AHI or respiratory disturbance index obtained by polysomnography, which integrate additional physiological signals, including nasal airflow and oxygen saturation.

JAWAC nevertheless offers several practical advantages for clinical investigation in children. It is non-invasive, can be performed at home under relatively ecological sleep conditions, and provides information on mandibular activity, respiratory effort, respiratory events, and sleep fragmentation. Studies evaluating mandibular activity during sleep have also reported relationships between JAWAC-derived parameters and selected polysomnographic measures [46,47]. These findings support its use as a complementary exploratory tool, but do not establish diagnostic accuracy for UARS or justify its use as a substitute for polysomnography.

Of particular clinical interest is the possibility of identifying children in whom repeated increases in respiratory effort, respiratory events, or sleep fragmentation warrant specialized respiratory, sleep, or otolaryngological assessment. JAWAC may therefore contribute to the clinical pathway by providing objective information that complements the daytime examination and parental sleep history and can be compared during longitudinal follow-up. When significant abnormalities are observed, appropriate specialist assessment, including polysomnography when indicated, remains essential.

### 4.7. Illustrative Clinical Example: Stepwise Application of the Clinical Assessment

**Figure 3:**
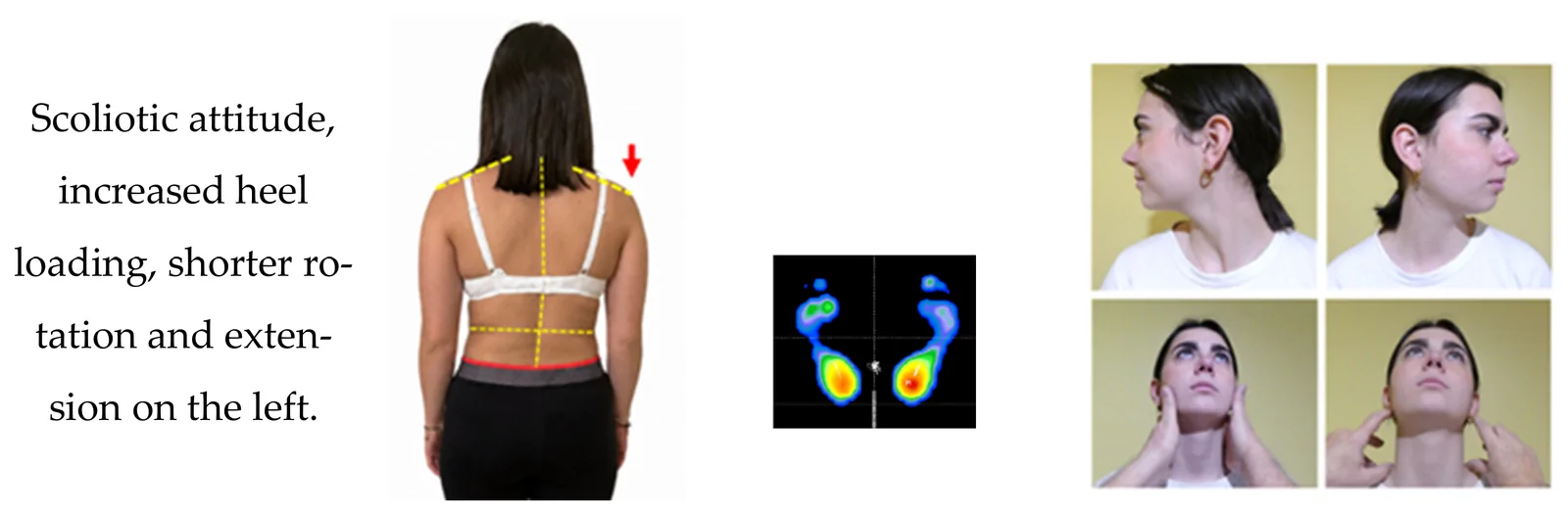

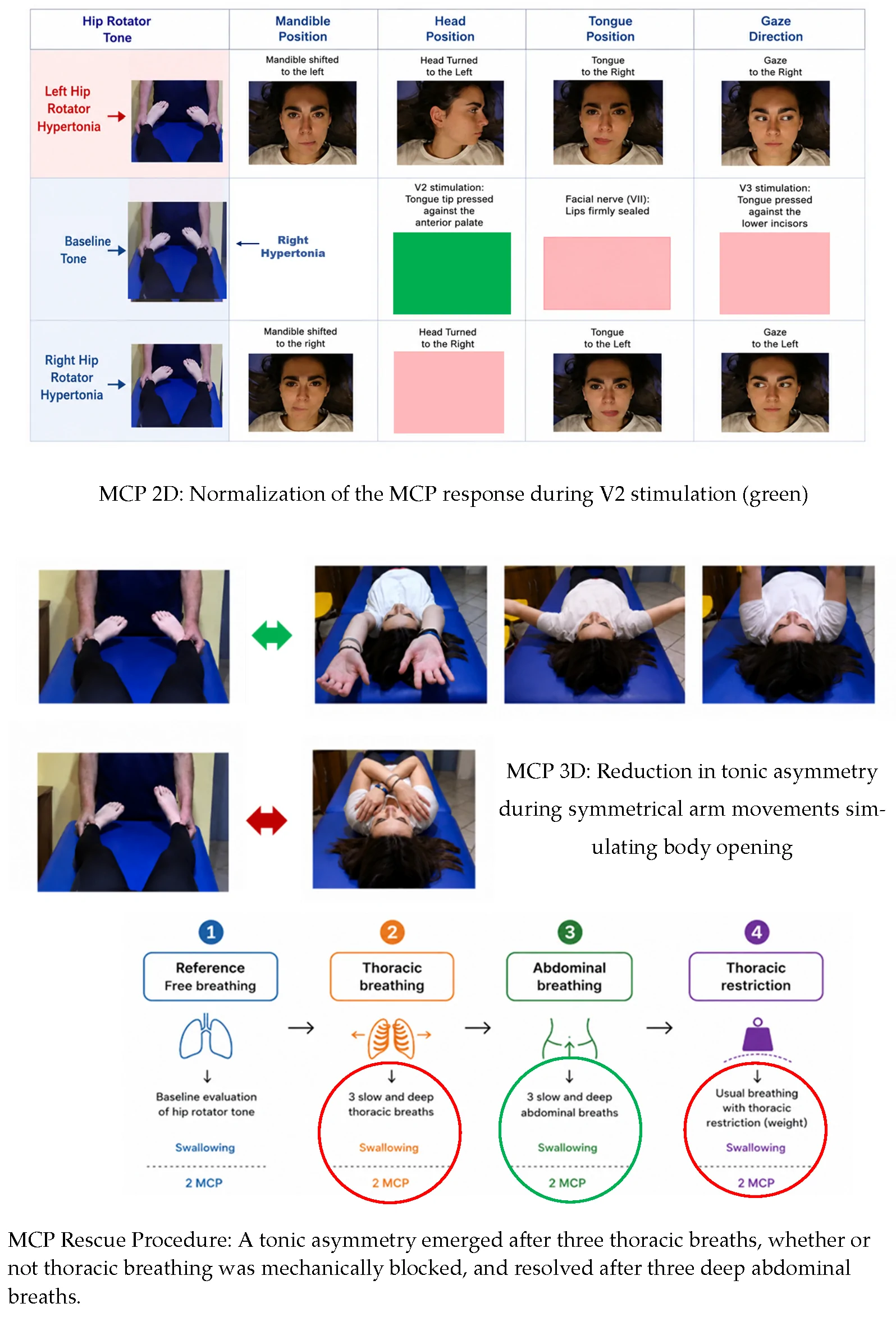

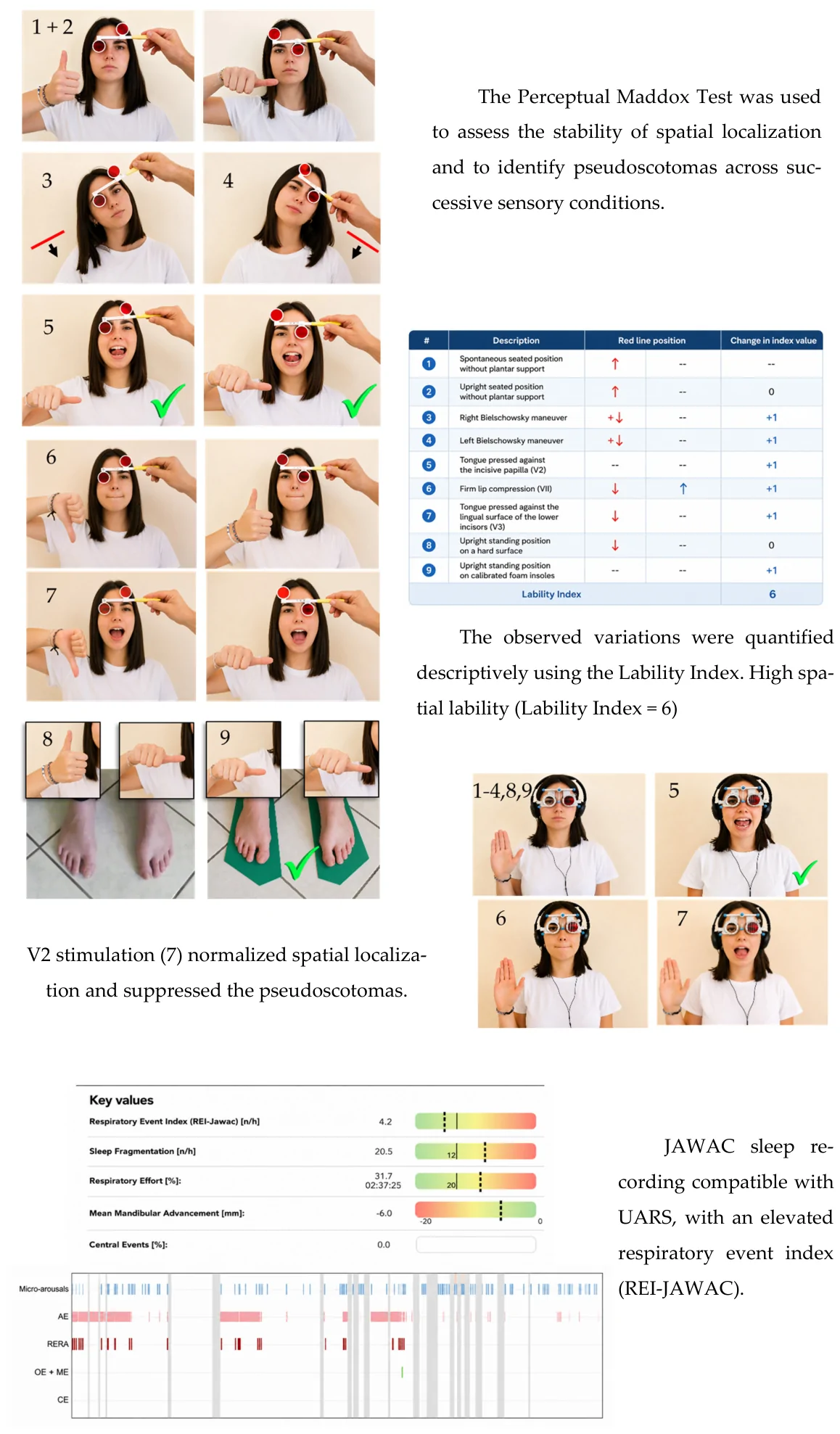
Sequence of the clinical assessment, from motor organization to spatial localization, multisensory integration, and sleep-related respiratory assessment.

This clinical case illustrates the stepwise application of the proposed assessment, from motor organization to spatial localization and multisensory integration (Figure 3). The examination identified a functional scoliotic posture and predominant heel loading, followed by a shorter range of cephalic rotation and extension on the left. During the MCP 2D, stimulation of the maxillary branch of the trigeminal nerve (V2) produced a regulatory response, with a reduction in the tonic asymmetry. During the MCP 3D, symmetrical upper-limb positions associated with greater body opening similarly reduced the tonic asymmetry. The Perceptual Maddox Test and pseudoscotoma assessment showed that V2 stimulation also produced the most consistent stabilizing effects on spatial localization and multisensory perception. Thus, across the different parts of the examination, V2 stimulation emerged as the sensory condition producing the most consistent regulatory responses and provided information that guided the subsequent management strategy. Greater body opening was subsequently selected as the main body-level modulation, with the aim of reducing the postural contribution of the diaphragm and favoring a predominantly respiratory role.

The subsequent sleep assessment using JAWAC showed marked nocturnal respiratory fragmentation, with frequent respiratory events and increased respiratory effort, a pattern compatible with increased upper-airway resistance and raising the possibility of UARS in this individual. The MCP Rescue further showed a tonic response during thoracic breathing restriction that was reduced after several correctly performed abdominal breaths. These observations were not interpreted as evidence of an immediate therapeutic effect. Rather, they provided additional clinical information that was incorporated into the subsequent assessment and contributed to the working hypothesis that respiratory and postural functions were functionally linked in this patient.

## 5. Sensorimotor Modulation: Different Sensory Gates Toward a Common Objective

The clinical examination described in the previous section is not intended merely to identify isolated abnormalities. A primary purpose is to determine which sensory conditions appear to improve, perturb, or leave unchanged the different dimensions of sensorimotor organization explored during the assessment.

The interventions described below have been developed within this clinical framework as forms of sensorimotor modulation. They are not presented as treatments for developmental dyslexia itself. Their objective is more limited and specific: to modify, when possible, the sensory and functional conditions in which the child operates during the day and, when relevant, during sleep.

The underlying clinical aim is to place the child in conditions that may be more favorable to attention, learning, and the consolidation of newly acquired skills. Sensorimotor modulation is therefore not intended to replace interventions that directly address reading, language, or learning difficulties. Rather, it is conceived as a complementary approach intended to support the functional conditions in which educational and rehabilitative interventions take place.

**Figure 4:**
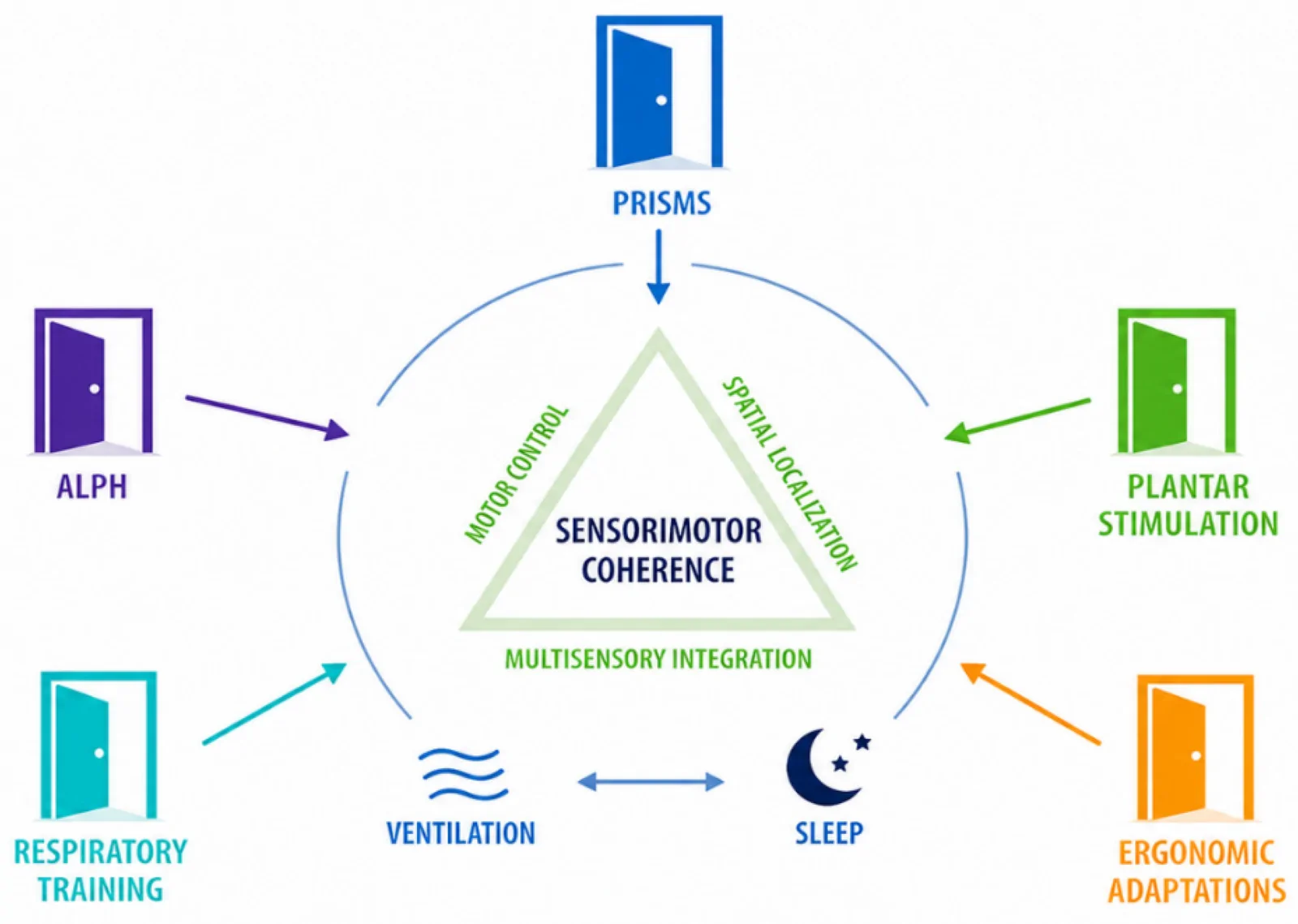
Different sensory gates, one common objective. The interventions described in this article act through different sensory and functional entry points. Their purpose is not to correct isolated dysfunctions, but to modify, when possible, sensory and physiological conditions associated with the individual’s sensorimotor organization. These approaches may therefore be considered complementary routes for testing and, when appropriate, maintaining a more stable functional response.

Although these interventions act through different mechanisms and sensory entry points, they share this common objective. Prisms, micrometric oral stimulations, proprioceptive insoles, respiratory rehabilitation, manual therapy, and ergonomic recommendations can therefore be viewed as different routes through which the clinician may attempt to modify the individual’s functional conditions (Figure 4).

The choice of a particular sensory gate does not depend solely on theoretical preference. It is guided by the responses observed during the clinical examination. In particular, the clinician considers whether a given sensory or functional modification produces a reproducible and favorable response within one or more functional domains, and whether this response is concordant with observations obtained elsewhere.

The objective is therefore not to normalize a specific sensory parameter or to claim correction of dyslexia through sensory intervention. Rather, it is to identify and, when possible, maintain sensory and nocturnal conditions that appear compatible with a more stable functional organization. Whether such modifications influence attention, learning, reading development, or sleep quality remains an empirical question requiring appropriate longitudinal and controlled investigation.

### 5.1. Ocular Modulation: Acting on Spatial Reference Frames

The oculomotor system occupies a particular position within sensorimotor organization. Extraocular muscles contribute not only to gaze control but also to sensory information involved in the construction of spatial reference frames.

The use of very low-power prisms in sensorimotor approaches can be traced back to the pioneering work of Baron, who reported immediate changes in axial muscle tone following prism application [49]. Subsequent experimental studies on ocular proprioception have provided a physiological framework in which spatial localization and visuospatial processing emerge from interactions between retinal information, oculomotor signals, and proprioceptive inputs from the extraocular muscles [31,50,51,52,53].

Within the present clinical framework, prisms are used not primarily to correct an ocular deviation, but to modify visual spatial information and observe the resulting sensorimotor response. Their purpose is to determine whether a defined modification of visual input contributes to a more stable organization of tonic regulation and spatial localization.

**Figure 5:**
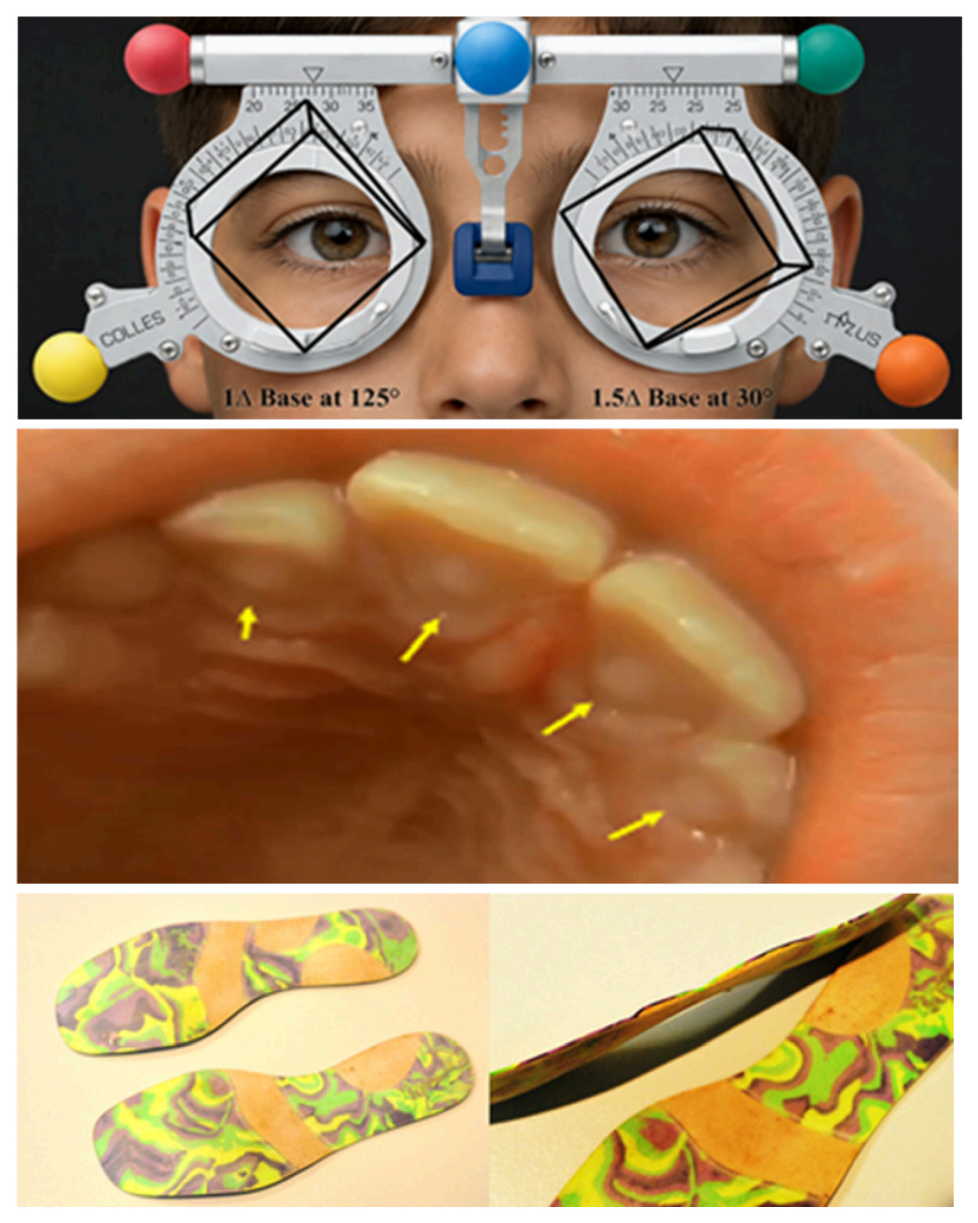
Individualized sensorimotor modulation in the clinical case: very low-power oblique prisms (Prisms of 1.0 prism diopter on the right and 1.5 prism diopters on the left), palatal ALPH stimulation (V2 stimulation) and proprioceptive insoles with localized plantar elements (very thin retrocapital elements and calcaneal wedges). Together, these interventions were selected to promote greater body opening and a more coherent sensorimotor organization across the three functional domains explored.

The prisms used in this approach are of very low power (Figure 5—image generated by ChatGPT-5.6; -OpenAI). They are prescribed bilaterally, generally with unequal powers, with their axes oriented according to the clinical findings. The selection of these parameters is individualized and is based on the responses observed during the clinical examination rather than on a single ocular measurement.

Prism power is primarily guided by the responses observed during the 3D MCP, particularly the changes in tonic asymmetry produced by symmetrical modifications of upper-limb proprioceptive input. The Perceptual Maddox Test then provides complementary information regarding the precise orientation of the prisms and the effect of the proposed configuration on spatial localization and its stability.

The final prescription is therefore established iteratively. A configuration is retained when its introduction produces reproducible and concordant effects across several functional domains, particularly tonic regulation, spatial localization, and multisensory responses. Conversely, a configuration that improves one domain while perturbing another may be reconsidered or further adjusted.

This approach illustrates a central principle of sensorimotor modulation: the prism is not considered an isolated corrective device, but one sensory gate within a broader therapeutic strategy. Its effects are therefore interpreted in relation to those produced by oral, plantar, respiratory, and, when indicated, manual interventions.

The objective is not to treat developmental dyslexia through prism correction itself. Rather, ocular modulation is intended to modify the sensory conditions under which the child organizes posture and spatial orientation and, when a favorable response is observed, to contribute to a more coherent overall sensorimotor organization.

### 5.2. Oral Trigeminal Modulation: ALPH^®^ (ALPH^®^: Microreliefs, Named from the First Names of Alfredo Marino and Philippe Villeneuve)

The oral cavity occupies a particular position within sensorimotor organization. Richly innervated by the trigeminal system, it contributes to mastication, swallowing, speech, and the sensorimotor organization of the upper airway [10,54]. Functional interactions between the tongue, mandible, hyoid bone, cervical musculature, and diaphragm provide a physiological basis for considering oral sensorimotor function as relevant to both posture and respiration [10,55].

ALPH consist of micrometric oral stimulations, typically a few hundred micrometres in thickness, placed on selected dental surfaces (Figure 5). Their purpose is not to correct an orthodontic abnormality or mechanically modify occlusion, but to modify sensory information arising from the oral and periodontal tissues through trigeminal afferent pathways [56,57].

Within the present therapeutic framework, particular attention is given to swallowing function. By providing a continuous oral sensory stimulus, ALPH are intended to influence the sensorimotor organization involved in swallowing. This characteristic distinguishes them from several other daytime interventions, as the stimulation remains present during sleep.

This nocturnal persistence is of particular interest in the present framework because swallowing is an automated function that interacts closely with upper-airway and respiratory organization. When oral stimulation is associated with a more appropriate swallowing pattern during wakefulness, it may also contribute to a more favorable organization of nocturnal respiratory function. This possibility remains a clinical hypothesis and requires systematic investigation.

The prescription of ALPH is individualized. Their location, thickness, and right–left distribution are adjusted according to the functional responses observed during the clinical examination, particularly when oral stimulation produces a reproducible change in the response being explored. The objective is not to reproduce a standardized dental configuration, but to identify an oral sensory input that is functionally appropriate for the individual child.

ALPH may be used alongside conventional orthodontic treatment. The two approaches pursue different objectives: orthodontic treatment primarily modifies anatomical relationships, whereas ALPH are intended to modify oral sensory input. Their possible complementarity should therefore be considered from a functional rather than purely anatomical perspective.

### 5.3. Plantar Modulation: Acting on Plantar Sensory Inputs

The foot constitutes one of the principal sensory interfaces between the body and its mechanical environment. Information arising from the plantar surface contributes continuously to the estimation of body orientation and verticality, the distribution of body weight, tonic regulation, and the postural adjustments required for balance maintenance.

The plantar surface contains a highly differentiated functional sensory map. As demonstrated by Kavounoudias and colleagues, the location of stimulation beneath the foot influences the postural response according to the region stimulated [58,59,60,61]. This precise spatial organization of plantar sensory receptors provides the basis for using very small, localized stimulations to modify the sensory information available to the postural system.

Within the present framework, proprioceptive insoles are therefore not primarily designed to modify the mechanical structure or morphology of the foot. Their main purpose is to modify plantar sensory input by selectively stimulating defined regions of the plantar surface. This distinction is important because a mechanical correction and a sensory modulation may coexist, but they pursue different objectives.

The sensitivity of plantar mechanoreceptors explains why the elements incorporated into the insoles can be extremely thin, generally ranging from a few tenths of a millimetre to approximately two millimetres (Figure 5) [62]. Small changes in the mechanical stimulation of selected plantar regions may consequently produce changes in sensory input and, potentially, in postural organization.

Postural observation and functional podiatric assessment provide the initial basis for selecting candidate areas of stimulation. The final configuration, however, is determined by the functional responses observed during the clinical examination. Different configurations may therefore be tested according to their effects on tonic organization, cephalic mobility, spatial localization, and multisensory responses.

The location, shape, thickness, and stiffness of the insole components are progressively adjusted according to the observed responses. The objective is not to achieve an ideal morphological correction, but to identify a plantar sensory configuration that is functionally appropriate for the individual child. A mechanical correction may be incorporated when clinically indicated, but it remains distinct from the sensory objective of the proprioceptive insole.

### 5.4. Manual Medicine: Removing Mechanical Obstacles to Sensorimotor Modulation

In some patients, persistent muscular hypertonia or restricted joint mobility may interfere with the effects of sensorimotor modulation. Under these conditions, the responses observed during the clinical examination may remain unstable, and the effects of sensory interventions may be difficult to reproduce or maintain.

During the clinical examination, particular attention is therefore paid to mechanical restrictions that may modify the responses obtained during the different functional tests. Temporary manual mobilization may sometimes be accompanied by immediate changes in these responses, such as improved tonic symmetry or greater perceptual stability. These changes are considered functional observations that may indicate that a mechanical restriction is interfering with the sensorimotor organization being explored.

The purpose of manual medicine in this framework is therefore not to treat developmental dyslexia directly, but to remove mechanical constraints that may limit the child’s capacity to adapt to sensory modulation. When such a constraint is identified, the child may be referred to an appropriately trained practitioner in manual medicine.

The manual treatment itself does not rely on a technique specific to the present approach, but on methods commonly used in manual medicine [63,64]. Its place within the present strategy lies in the integration of these interventions with the broader sensorimotor assessment. After the mechanical restriction has been addressed, the sensory modulations previously identified can be finely adjusted and their functional effects reassessed.

This approach is therefore consistent with the general principle of the present treatment strategy: rather than treating a symptom directly, the clinician seeks to create conditions in which a more stable sensorimotor organization can emerge.

### 5.5. Ergonomic Adaptations: Supporting Sensorimotor Stability During Learning

The objective of ergonomic adaptations is not merely to improve comfort during schoolwork. Within the present framework, they are intended to provide working conditions that support a more stable sensorimotor organization during prolonged reading and writing activities.

**Figure 6:**
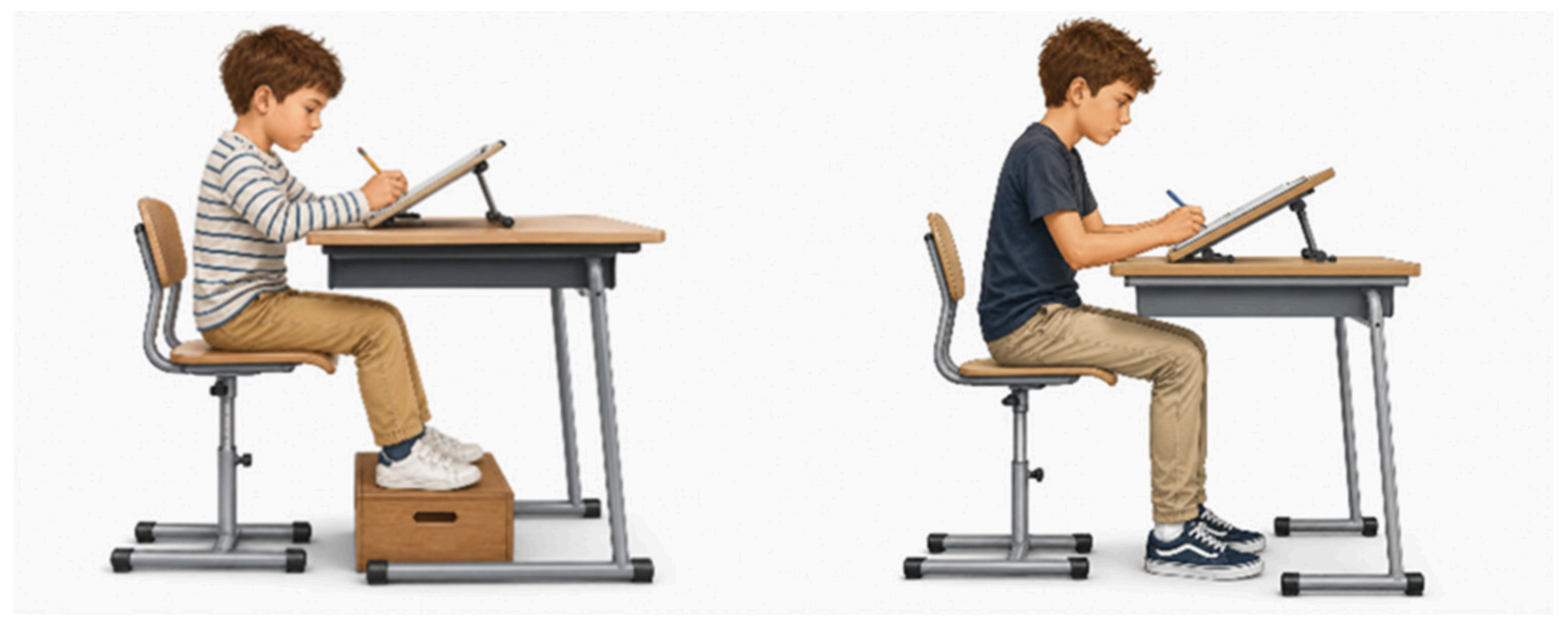
Two working positions used in clinical practice, selected according to the child’s height and the available equipment.

Particular attention is paid to the position adopted at the workstation. The child is encouraged to work in a configuration that promotes postural symmetry and minimizes unnecessary mechanical constraints [65,66]. This can be achieved through simple adjustments of the seating position, desk height, and lower-limb support (Figure 6—generated by ChatGPT-5.6; -OpenAI).

A sloping work surface, inclined approximately 20–30°, is systematically recommended. In addition to its practical advantages for reading and writing, this configuration may reduce some of the postural and oculomotor demands associated with prolonged downward gaze.

Adequate support of the feet is equally important. Although plantar cutaneous afferents are classically associated with standing balance, they remain active in the seated position and contribute to sensory information concerning body orientation [67]. Whenever possible, both feet should therefore remain in stable contact with a support surface, either directly on the floor or through a footrest according to the child’s height.

The ergonomic configuration is adapted to the child’s anthropometric characteristics and to the sensory-motor responses observed during the clinical examination. These adjustments are simple, but they aim to reduce environmental constraints that could interfere with the maintenance of sensorimotor stability during sustained learning activities.

These measures do not constitute a treatment for developmental dyslexia in themselves. Rather, they complement the different sensorimotor modulation strategies described above by creating working conditions that are more compatible with the child’s individual sensorimotor organization.

The next stage of treatment focuses on another major component of sensorimotor regulation: respiratory function. Unlike ocular, trigeminal, and plantar interventions, respiratory rehabilitation acts on a function positioned at the intersection of posture, sleep, and sensorimotor regulation.

### 5.6. Respiratory Rehabilitation, Sleep, and Brain Plasticity

Improving sensorimotor coherence does not depend solely on the modifications achieved during wakefulness. It also requires adequate nocturnal recovery, during which sleep contributes to memory consolidation, learning, and brain plasticity.

Our clinical experience progressively led us to consider respiratory function during sleep as a central component of the therapeutic strategy. Information obtained from the clinical history, the Rescue MCP, and objective sleep assessment helps identify children in whom respiratory function may interfere with nocturnal recovery.

Within the present framework, particular attention is given to the functional role of the diaphragm. When postural demands are chronically associated with diaphragmatic activity, the diaphragm may participate excessively in postural stabilization and insufficiently in its primary respiratory function. This may be particularly relevant during REM sleep, when the activity of intercostal and accessory respiratory muscles is markedly reduced and ventilation becomes predominantly dependent on diaphragmatic function.

The sensory modulations described above aim in part to reduce these postural constraints. However, in our experience, this alone is often insufficient to obtain a clear and sustained improvement in nocturnal sleep quality. Recovery of an efficient diaphragmatic breathing pattern therefore represents a central component of the therapeutic strategy.

**Figure 7:**
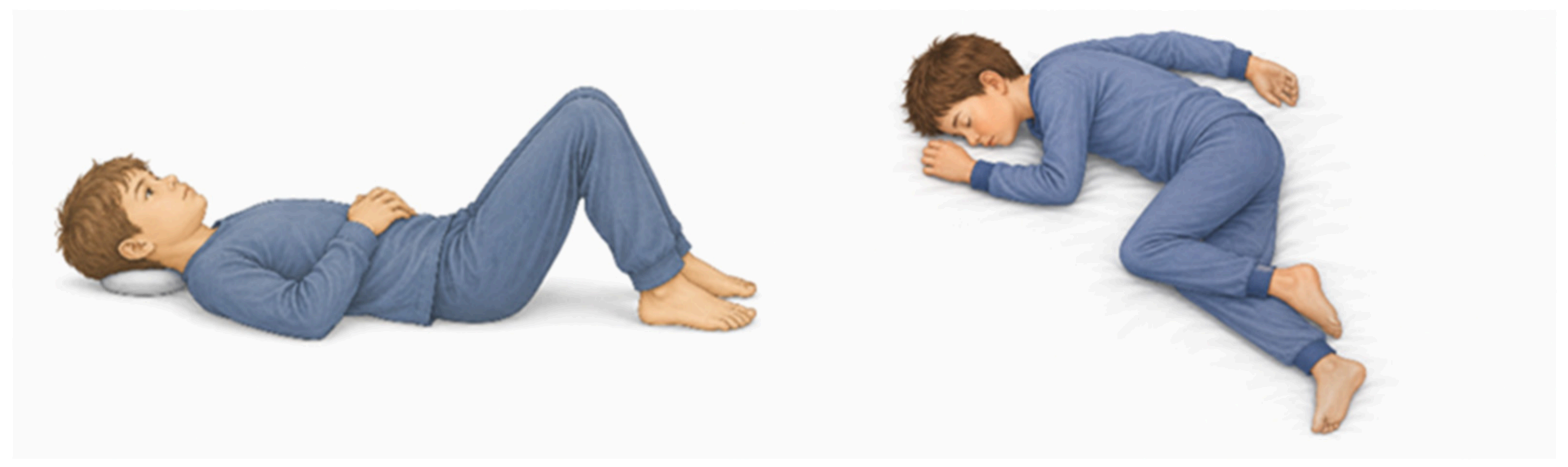
Recommended positions for practicing diaphragmatic breathing before bedtime and for initiating sleep. The latter position is intended to facilitate maintenance of an appropriate respiratory pattern during sleep (generated by ChatGPT-5.6; -OpenAI).

Respiratory rehabilitation is designed to progressively restore this function. It is based primarily on the acquisition of efficient diaphragmatic breathing, practiced daily, with particular emphasis on the period immediately before sleep onset. Exercises are taught to families using simple video-based instructions and are repeated over several months to facilitate the gradual automation of the new respiratory pattern (Figure 7—generated by ChatGPT-5.6; -OpenAI) [68,69].

This timing is considered particularly important because it may facilitate the transition from the respiratory pattern practiced during wakefulness toward a more automatic breathing pattern during sleep. This is relevant because sleep provides the physiological conditions required for memory consolidation, neuroplasticity, recovery, and next-day cognitive functioning.

In clinical practice, this stage may represent one of the most challenging components of treatment. Many children have already undergone several rehabilitation programs and may understandably be reluctant to undertake additional daily exercises. Parental involvement is therefore particularly important. Regular practice appears to be an important determinant of the stability of the respiratory adaptation achieved.

Our experience suggests that children who perform the exercises regularly generally achieve more consistent improvements, whereas poor adherence is frequently associated with more limited changes despite appropriate use of sensory modulation devices. This observation further emphasizes the importance of family participation in the therapeutic process.

The results obtained during our exploratory intervention study support this approach. Parent-reported sleep disturbances showed a highly significant improvement on the Sleep Disturbance Scale for Children (SDSC; *p* < 0.001), whereas 3D actigraphy did not detect a significant change in nocturnal motor activity. No polysomnographic respiratory recording was performed in this intervention study. Improvements were also observed in attentional flexibility and in several reading-performance measures.

Because the intervention combined several components, including proprioceptive modulation, speech therapy, and bedtime breathing exercises, these findings do not establish a specific causal effect of respiratory rehabilitation on sleep or learning. They nevertheless support the hypothesis that improving sleep-related physiological conditions may contribute to the consolidation of daytime adaptations.

Within this framework, respiratory rehabilitation therefore has a broader objective than improving ventilation alone. It aims to restore the conditions in which sleep can fulfil its physiological functions of recovery, memory consolidation, and neuroplasticity.

Sleep is consequently not considered a secondary aspect of treatment. It is an integral component of the therapeutic strategy. The daytime interventions seek to modify the child’s sensory conditions for action and learning, while respiratory rehabilitation seeks to ensure that these adaptations can be supported and consolidated during sleep.

## 6. A Proprioception-Centered Hypothesis Linking Sensorimotor Function, Respiration, and Sleep Across the Day–Night Cycle

### 6.1. An Approach Based on Sensorimotor Adaptation

The observations presented in this article suggest that the motor control characteristics observed in some children with developmental dyslexia may be better understood as adaptive organizations rather than fixed abnormalities. The changes observed following different forms of sensorimotor modulation suggest that these organizations retain a substantial capacity for functional reorganization. Within the framework proposed here, proprioception occupies a central position in this adaptive organization, contributing to the coherence between sensory information, motor regulation, and body-centered spatial representations. This adaptability constitutes one of the foundations of the clinical approach described here.

Within this perspective, the therapeutic strategies described above are not directed toward isolated symptoms or toward a single sensory modality. Their purpose is to modify the sensory and functional conditions under which posture, spatial localization, multisensory integration, respiration, and sleep are organized. Proprioception is therefore not considered as one sensory modality among others, but as an organizing component around which these different functions may interact. Because these functions interact, the objective is not simply to obtain a favorable response to each intervention considered separately, but to identify sensory modulations that are mutually compatible and contribute to a coherent organization across functional domains. Prisms, micrometric oral stimulation (ALPH), proprioceptive insoles, respiratory rehabilitation, manual therapy, and ergonomic adaptations therefore represent complementary sensory or functional gates within a single coordinated strategy.

During wakefulness, this strategy seeks to create conditions compatible with stable posture, appropriate spatial orientation, coherent multisensory processing, attention, and learning. During sleep, it seeks to preserve conditions favorable to respiratory function and restorative sleep, thereby allowing sleep-dependent processes of memory consolidation, brain plasticity, and next-day functioning to take place. These two objectives are not independent: the aim is to establish continuity between daytime sensorimotor adaptation and nighttime physiological recovery, while avoiding a sensory modulation that improves one function at the expense of another.

This perspective progressively led us beyond a model centered on isolated sensory abnormalities toward a broader framework emphasizing the adaptive properties of sensorimotor organization. The observations presented throughout this article suggest that some of the functional disturbances identified during clinical assessment may be related to proprioceptive mechanisms involved in the organization of sensorimotor coherence across the day–night cycle.

### 6.2. Proprioception-Centered Sensorimotor Coherence and the Conditions for Plasticity

The observations described throughout this article suggest possible relationships between several domains that are often studied separately, including sensorimotor regulation, posture, respiration, sleep, attention, and learning.

Several of these relationships are supported by observations reported in the literature. Developmental dyslexia may coexist with motor coordination difficulties, language disorders, attentional difficulties, and sleep disturbances. Motor abnormalities have been investigated in dyslexia research, while sleep-disordered breathing has been associated with attentional and learning difficulties in several populations [70,71,72,73,74,75]. Prematurity, which is associated with both sleep-related respiratory disturbances and an increased prevalence of learning disorders, provides another example of this convergence [76,77]. Together, these observations raise the possibility that some of the difficulties observed in developmental dyslexia may develop within a broader physiological context involving sensorimotor regulation across the day–night cycle.

Within the clinical framework presented here, proprioceptive mechanisms occupy a central position. Our observations suggest that disturbances affecting the construction and use of body-centered spatial reference frames may be associated with adaptive changes involving posture, respiration, and sleep. The direction of these relationships cannot be determined from the present observations alone: such disturbances may represent an early developmental component, a consequence of other developmental factors, or one element of a reciprocal interaction.

The proposed framework does not place proprioception outside established developmental accounts of dyslexia. Rather, proprioceptive sensorimotor organization is considered one functional level through which different developmental factors may interact with motor regulation, respiration, sleep, attention, and learning.

From this perspective, motor disturbances reported in developmental dyslexia may not necessarily represent independent comorbidities. In some children, they may form part of an adaptive sensorimotor organization that interacts with respiratory and sleep-related functions. Conversely, sleep disturbances may influence motor regulation, attention, and plasticity. The resulting organization may therefore reflect reciprocal interactions rather than a simple linear causal chain.

This perspective extends the embodied framework proposed in our previous work [25]. Rather than considering cognition independently of the bodily conditions in which it develops, it emphasizes potential interactions linking body representation, action, respiration, sleep, and adaptive plasticity across the day–night cycle.

Such a framework may also help conceptualize the clinical heterogeneity observed among children with developmental dyslexia. If different children develop different adaptive responses to partially overlapping developmental constraints, diverse sensorimotor organizations may emerge. This interpretation remains hypothetical and requires prospective investigation.

The observations presented in this article therefore lead us to formulate the following hypothesis: proprioception-centered sensorimotor coherence may constitute one functional organizer through which motor, respiratory, attentional, and learning-related functions interact during development.

Within this framework, sensorimotor modulation is therefore conceived not as a direct treatment for dyslexia, but as an attempt to create physiological and functional conditions that may support adaptive and learning processes.

### 6.3. Sleep: An Essential Phase in Understanding the Role of Proprioception-Centered Sensorimotor Coherence in Learning

Understanding the role of proprioception-centered sensorimotor coherence in learning requires consideration not only of daytime sensorimotor organization, but also of the quality of the sleep that follows.

Sleep plays a fundamental role in memory consolidation, procedural learning, attentional regulation, and brain plasticity [78,79]. At the same time, repeated clinical observations of paradoxical breathing patterns, nocturnal restlessness, morning fatigue, and sleep-disordered breathing among children with developmental dyslexia led us to explore more closely the interactions linking posture, respiration, sleep, and learning.

This perspective progressively broadened our understanding of sensorimotor regulation. The adaptations observed during the day cannot be considered independently of the processes of recovery and consolidation occurring during sleep. Within the framework proposed here, daytime sensorimotor organization and nighttime recovery are therefore viewed as functionally connected, with the quality of sleep potentially influencing the extent to which daytime adaptations can be consolidated.

Within this framework, sensory modulations performed during the day are not viewed as an end in themselves. Their potential value also lies in their capacity to create physiological conditions compatible with restorative sleep, memory consolidation, attentional regulation, and brain plasticity.

The importance attributed to sleep therefore does not imply that the sleep–wake cycle itself constitutes a pathological mechanism. Rather, the present hypothesis proposes that the quality of nocturnal sleep may represent a critical condition through which daytime sensorimotor adaptations can support subsequent recovery, consolidation, and learning. Daytime sensorimotor modulation and nighttime physiological recovery may thus be considered complementary components of a broader adaptive process unfolding over time.

From this perspective, understanding the child’s sensorimotor organization requires consideration of both sides of this functional sequence: the sensory and postural conditions in which the child acts and learns during the day, and the physiological conditions under which these experiences are subsequently consolidated during sleep. This interpretation remains part of the proposed integrative framework rather than a demonstrated causal mechanism.

### 6.4. From Clinical Observations to Preliminary Intervention Studies

The clinical observations that gave rise to the present framework also led progressively to exploratory intervention studies. An early study published in 2007 evaluated a proprioceptive and postural intervention based on prismatic correction in children with developmental dyslexia [80]. Some reading-related measures improved during follow-up, whereas changes in phonological performance were less consistent. Although limited, these findings provided the initial basis for a nearly 20-year program of fundamental research in our INSERM laboratory, aimed at objectively investigating the sensorimotor phenomena observed in clinical practice.

In parallel with this transition toward fundamental research, clinical practice continued to explore how sensory modulations could be used to modify functional responses. The progressive repetition of these clinical observations led to increasingly precise characterization of the conditions under which ocular, oral, plantar, respiratory, and postural modulations produced regulatory, neutral, or perturbing effects. Thus, the research program progressively generated a body of experimental and clinical data in parallel, while the clinical procedures used to select and adjust sensory modulations became increasingly refined.

Over all these years, studies conducted in our laboratory examined postural regulation, proprioceptive acuity, spatial localization, multisensory integration, and motor representation in developmental dyslexia [16,17,18,19,20,21,22,23,24,29]. These studies provided objective measures of several phenomena that had initially been identified primarily through clinical observation.

More recently, the extension of this research program to respiration and sleep led to exploratory clinical studies combining proprioceptive modulation with speech therapy and breathing exercises practiced immediately before sleep onset [24]. These studies reported changes in sleep-related symptoms, attentional flexibility, and reading performance [24]. In parallel, an independently conducted randomized controlled trial reported improvements in silent reading performance and eye-movement organization after the addition of a proprioceptive intervention to conventional speech therapy [81].

Taken together, these studies illustrate the progressive development of the research program rather than a predefined theoretical sequence: clinical observations generated experimental questions; fundamental research provided objective measures of selected phenomena; in parallel, continued clinical exploration progressively refined the characterization and selection of sensory modulations; and subsequent exploratory interventions extended the investigation toward functional outcomes. The available findings therefore contribute to the development of the present hypothesis without, by themselves, establishing its causal model or the efficacy of its individual therapeutic components.

### 6.5. Limitations and Perspectives

The framework presented in this article should be considered a working hypothesis rather than an established causal model of developmental dyslexia. Its different components are supported by evidence of different levels and should therefore not be interpreted as having equivalent evidential status.

First, the available evidence does not establish that proprioceptive dysfunction is a primary cause of developmental dyslexia. Nevertheless, several experimental studies have shown that proprioceptive manipulations can influence postural regulation, spatial localization, visuospatial processing, and perceptual responses in children with dyslexia [16,17,18,19,20,21,22,23,24,29]. These findings indicate that proprioceptive information participates in functions relevant to the present framework, but they do not by themselves establish where proprioceptive mechanisms are situated within the causal pathways involved in developmental dyslexia.

Second, some of the relationships described in the clinical examination remain observational and mechanistically unresolved. Changes in tonic regulation, spatial localization, multisensory perception, respiration, and sleep may occur in association, but the mechanisms linking these observations remain to be determined. In particular, the respective contributions of proprioceptive, vestibular, visual, trigeminal, and other multisensory mechanisms cannot currently be fully separated. The present framework therefore provides a way of organizing these observations rather than a definitive explanation of their origin.

Developmental dyslexia is also a heterogeneous neurodevelopmental condition involving multiple interacting factors. The present framework is consequently not proposed as an alternative to cognitive, linguistic, phonological, or attentional models. Rather, it proposes that sensorimotor organization may constitute an additional level of analysis, particularly for understanding potential interactions among posture, spatial representation, multisensory processing, respiration, sleep, and the physiological conditions supporting learning.

An important characteristic of the present approach is its clinical origin. The framework developed progressively from repeated observations in clinical practice, which subsequently generated experimental questions. Experimental studies were then used to investigate whether phenomena initially identified clinically could be objectively measured and characterized. In parallel, continued clinical exploration progressively refined the characterization and selection of sensory modulations. This sequence differs from the conventional development of a treatment from a previously established mechanistic model and explains why some components of the approach remain insufficiently validated despite more than two decades of clinical and experimental investigation.

The same consideration applies to the therapeutic interventions. The available evidence is not sufficient to establish proprioceptive modulation as a validated treatment for developmental dyslexia. Most intervention studies have involved multimodal interventions, relatively small samples, or exploratory designs, making it difficult to determine the specific contribution of individual components such as ocular, oral, plantar, respiratory, or manual interventions. The present article therefore does not propose proprioceptive modulation as a direct treatment for dyslexia. Its proposed objective is instead to modify the sensory and physiological conditions in which the child learns, with particular attention to sensorimotor organization and sleep-dependent recovery and plasticity.

This distinction is important when interpreting the clinical observations reported here. Improvements observed following sensorimotor modulation should not be interpreted as proof that the underlying mechanisms of developmental dyslexia have been corrected. They indicate, rather, that some functional responses relevant to sensorimotor organization may remain modifiable. Whether such modifications translate into reproducible and clinically meaningful improvements in learning requires further investigation.

Future studies should therefore examine the different components of the framework separately as well as in combination. Larger controlled and multicenter studies, incorporating objective measures of sensorimotor function, sleep, attention, and learning, will be necessary to determine the magnitude, specificity, durability, and reproducibility of the observed effects. Such studies should also help determine whether particular clinical profiles are more likely to respond to specific forms of sensorimotor modulation.

The broader perspective proposed here is therefore deliberately testable and open to revision. If future research confirms some of the relationships described in this article, proprioception-centered sensorimotor coherence may prove to be one of the functional frameworks through which posture, perception, respiration, sleep, plasticity, and learning interact during development. If some components are not confirmed, the framework will need to be refined accordingly.

The value of the present framework therefore lies not in claiming a final explanation of developmental dyslexia, but in proposing a coherent set of clinically observable and experimentally testable relationships that may help connect domains that have traditionally been studied separately. Taken together, the convergence of clinical observations and experimental findings provides sufficient rationale for further investigating the potential role of proprioception-centered sensorimotor coherence in developmental dyslexia and for determining whether this dimension should be incorporated into future integrative models of the disorder.

## 7. Conclusions

The approach presented in this article originated from a clinical observation: the unusually frequent occurrence of motor control disturbances in children with developmental dyslexia.

Over time, this initial observation led to the exploration of increasingly diverse domains, including proprioception, postural regulation, spatial localization, multisensory integration, respiration, and sleep. What progressively emerged was not a series of isolated findings, but a clinical and experimental perspective in which these different functions can be considered in relation to one another.

At the center of this perspective is the hypothesis that proprioception-centered sensorimotor coherence may represent one functional level through which body organization, perception, respiration, attention, and learning interact across the day–night cycle during development.

This perspective also provides a common framework for the different clinical approaches described in this article. Ocular, oral, plantar, respiratory, manual, and ergonomic interventions are not presented as independent treatments, but as complementary ways of modifying sensory or functional conditions according to the responses observed in each child.

The clinical objective is consequently not to correct a single abnormal parameter, but to identify the sensory and functional conditions associated with a more coherent sensorimotor organization. Such an approach may help provide a more favorable context in which educational, linguistic, and rehabilitative interventions can operate.

More broadly, this work suggests that understanding developmental dyslexia may benefit from considering not only the mechanisms directly involved in reading, but also the bodily and physiological context in which development, learning, recovery, and consolidation take place. The convergence of the clinical and experimental observations presented here provides a rationale for continuing to investigate these interactions and for determining the place that proprioception-centered sensorimotor coherence may occupy within broader integrative models of developmental dyslexia.

## Figures and Tables

**Table 1:** Questionnaire Assessing Daytime and Nighttime Signs of Dysproprioception.

**For each symptom, indicate the number that corresponds to the frequency:** **1 = ** **Never, 2 = Occasionally (1 or 2 times a month), 3 = Sometimes (1 or 2 times a week), 4 = Often (3 to 5 times a week), 5 = Every day.**	
**15 Questions for PARENTS** (after observing the child’s sleep for at least 3–4 h)	**SCORE**
The child jerks or moves parts of the body when falling asleep.	
The child has vivid, dream-like scenes when falling asleep.	
The child moves his legs a lot during sleep, often changes position during the night, or kicks off the bed covers.	
You have observed that your child sleepwalks.	
Your child has nightmares (terrors) that he cannot remember the next morning.	
He has great difficulty waking up in the morning.	
The child feels unable to move and is very tired when waking up in the morning.	
The child is sleepy during the day (for example, falls asleep easily in the car or when sitting quietly).	
The child salivates a lot at night, or there are traces of drool on the pillow in the morning.	
The child complains of a headache in the morning.	
The child breathes with their mouth open while sleeping.	
The child still wets the bed or often gets up at night to go to the toilet.	
The child has trouble remembering lessons learned the night before (even though they knew them the night before).	
The child sometimes seems a little sleepy at school.	
The child has an abnormal head position while sleeping (head tilted back and in extension).	
**19 Questions for the CHILD**	
**Muscular dimension**	
Do you feel tired even if you have not done any physical or mental activity?	
Is it hard for you to stand still and do nothing?	
Do you get a headache after school?	
Do you have repeated pain in your lower or upper back?	
Do your legs ever hurt?	
Is it hard for you to look at a text (or a person) up close?	
Do you ever see double when you are tired or after reading a text?	
Do you get out of breath quickly when you exercise (for example, as soon as you run)?	
Do you have blurred vision when looking at things up close after reading a few lines (with your glasses, if you have them)?	
**Spatial dimension**	
Is it difficult for you to walk on something narrow (for example, a beam)?	
Is it difficult for you to catch an object the first time (for example, a ball)?	
Do you fall easily or twist your ankles?	
Do you bite your tongue or cheeks when you eat?	
Do you bump into simple obstacles (for example, doorframes) as if you did not see the space around you properly?	
**Perceptive dimension**	
Do you feel like you are reading without understanding what you read?	
Do you find it hard to concentrate for a long time?	
When someone speaks to you, do you feel like you do not really understand what you hear?	
When you read, do you feel that you cannot see properly: you skip words or lose your place between lines, …	
Do you find it hard to express an idea when you are speaking, or to put your sentences together properly?	
**TOTAL SCORE—Enter the SUM of the scores for all 34 questions**	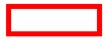

## Data Availability

No new data were created or analyzed in this study. Data sharing is not applicable to this article.

## Supplementary Materials

The following supporting information can be downloaded at: https://www.mdpi.com/article/10.3390/brainsci16101106/s1, Video S1: Perceptual Maddox Test.
